# The HCN Channel Blocker ZD7288 Induces Emesis in the Least Shrew (*Cryptotis parva*)

**DOI:** 10.3389/fphar.2021.647021

**Published:** 2021-04-29

**Authors:** W. Zhong, N. A. Darmani

**Affiliations:** Department of Basic Medical Sciences, College of Osteopathic Medicine of the Pacific, Western University of Health Sciences, Pomona, CA, United States

**Keywords:** HCN channel, ZD7288, emetic nuclei, calcium, emesis, least shrew

## Abstract

Subtypes (1–4) of the hyperpolarization-activated cyclic nucleotide-gated (HCN) channels are widely expressed in the central and peripheral nervous systems, as well as the cells of smooth muscles in many organs. They mainly serve to regulate cellular excitability in these tissues. The HCN channel blocker ZD7288 has been shown to reduce apomorphine-induced conditioned taste aversion on saccharin preference in rats suggesting potential antinausea/antiemetic effects. Currently, in the least shew model of emesis we find that ZD7288 induces vomiting in a dose-dependent manner, with maximal efficacies of 100% at 1 mg/kg (i.p.) and 83.3% at 10 µg (i.c.v.). HCN channel subtype (1–4) expression was assessed using immunohistochemistry in the least shrew brainstem dorsal vagal complex (DVC) containing the emetic nuclei (area postrema (AP), nucleus tractus solitarius and dorsal motor nucleus of the vagus). Highly enriched HCN1 and HCN4 subtypes are present in the AP. A 1 mg/kg (i.p.) dose of ZD7288 strongly evoked c-Fos expression and ERK1/2 phosphorylation in the shrew brainstem DVC, but not in the in the enteric nervous system in the jejunum, suggesting a central contribution to the evoked vomiting. The ZD7288-evoked c-Fos expression exclusively occurred in tryptophan hydroxylase 2-positive serotonin neurons of the dorsal vagal complex, indicating activation of serotonin neurons may contribute to ZD7288-induced vomiting. To reveal its mechanism(s) of emetic action, we evaluated the efficacy of diverse antiemetics against ZD7288-evoked vomiting including the antagonists/inhibitors of: ERK1/2 (U0126), L-type Ca^2+^ channel (nifedipine); store-operated Ca^2+^ entry (MRS 1845); T-type Ca^2+^ channel (Z944), IP_3_R (2-APB), RyR receptor (dantrolene); the serotoninergic type 3 receptor (palonosetron); neurokinin 1 receptor (netupitant), dopamine type 2 receptor (sulpride), and the transient receptor potential vanilloid 1 receptor agonist, resiniferatoxin. All tested antiemetics except sulpride attenuated ZD7288-evoked vomiting to varying degrees. In sum, ZD7288 has emetic potential mainly via central mechanisms, a process which involves Ca^2+^ signaling and several emetic receptors. HCN channel blockers have been reported to have emetic potential in the clinic since they are currently used/investigated as therapeutic candidates for cancer therapy related- or unrelated-heart failure, pain, and cognitive impairment.

## Highlights

The HCN channel blocker ZD7288 is pro-emetic in the least shrew.
ZD7288 evokes c-Fos expression in serotonergic neurons of the brainstem emetic loci.
ERK1/2 activation is involved in ZD7288-induced vomiting.
Ca2+ channel modulators reduce ZD7288-induced vomiting to varying degrees.
Serotonin 5-HT3 and neurokinin NK1 receptor antagonists attenuate ZD7288-evoked vomiting.

## Introduction

Defensive processes such as nausea and vomiting protect vomit-competent species avoid ingestion of toxic substances. While the act of forceful expulsion of gastrointestinal content through the mouth is called vomiting (or emesis), nausea is a painless unpleasant sensation that one may soon vomit. Vomiting may be preceded by retching behavior, where the gastrointestinal content is forced into the esophagus, but without the vomitus being expelled (Navari and Aapro, 2016; Adel, 2017). The anatomical sites involved in emesis include the dorsal vagal complex (DVC) emetic nuclei contained in the brainstem [the area postrema (AP), nucleus tractus solitarius (NTS) and dorsal motor nucleus of the vagus (DMNX)], the enteric nervous system (ENS) and enterochromaffin cells of the gastrointestinal tract, vagal afferents which carry emetic input from the gastrointestinal tract to the brainstem NTS, as well as vagal efferents which project motor signals from the DMNX to the gastrointestinal tract (Darmani and Ray, 2009; Babic and Browning, 2014). The AP and the NTS have loose capillaries, which allow diffusion of some circulating chemicals into the brainstem (Borison, 1989).

Hyperpolarization-activated cyclic nucleotide-gated (HCN) channels are a class of voltage-gated ion-channels permeable to Na^+^ and K^+^ and constitutively open at voltages near the resting membrane potential (Benarroch, 2013; McGovern et al., 2014). They consist of four subtypes 1–4, and are expressed on excitable cells in the central and peripheral nervous systems. The cation current (*I*_h_) mediated by HCN channels elicits membrane depolarization toward threshold for action potential generation, which plays a pivotal role in controlling neuronal excitability (Gill et al., 2004; Benarroch, 2013). In the brainstem AP, up to 60% of neurons express HCN channels (Shinpo et al., 2012). ZD7288 is considered as a specific HCN channels blocker that acts nonselectively among the four known HCN channels (Chaplan et al., 2003). ZD7288 can depress the excitability of the AP since it blocks HCN channel activation (Shinpo et al., 2012). Moreover, ZD7288 at very low doses (10^−3^–10^−4^ mg/kg, i. p.) can suppress apomorphine-induced conditioned taste aversion to saccharin preference and the apomorphine-evoked c-Fos expression in the rat area postrema (Shinpo et al., 2012). Based on these findings, the authors had suggested antinausea/antiemesis potential of ZD7288. However, they also found ZD7288 itself (>1 mg/kg) was the unconditioned stimulus for conditioned taste aversion learning (Shinpo et al., 2012), implying ZD7288 could be proemetic in emesis-competent species. In the clinic ivabradine was the first approved HCN4 blocker to lower heart rate and is currently used for the therapy of stable angina pectoris (Postea and Biel, 2011) and treatment of cancer chemotherapy-evoked left ventricular dysfunction (Sarocchi et al., 2018). Among patients receiving ivabradine, 3.3% have reported nausea (Chaturvedi et al., 2013).

Here in the present study, we sought to determine the emetic potential of the widely investigated pharmacological tool, the pyridinium derivative HCN channel blocker ZD7288, and its possible mechanisms. At First, we investigated whether systemic injection of varying doses of ZD7288 and ivabradine, two structurally different HCN channel blockers, can evoke vomiting in the least shrew animal model of vomiting. The emetic capacity of central microinjection of ZD7288 was also studied. Secondly, we examined the central and peripheral involvement of emetic loci underlying a fully effective emetic-dose of ZD7288 (1 mg/kg, i. p.) by means of c-Fos (Zhong et al., 2016) immunohistochemistry to indicate whether ZD7288 activates the brainstem DVC area or the ENS in the jejunum, or both. Double staining of brainstem sections with c-Fos and tryptophan hydroxylase 2 (TPH2), an enzyme invoved in 5-HT synthesis and a serotonergic neuronal marker (Cai et al., 2014), were utilized to determine whether serotonergic neurons are activated in response to ZD7288. Thereafter, we investigated the neurotransmitter basis of ZD7288-evoked vomiting via the use of several well-investigated receptor-selective antiemetics including: 1) ERK1/2 inhibitor U0126 (Hotokezaka et al., 2002), 2) L-type Ca^2+^ channel (LTCC) antagonist nifedipine (Triggle, 2007), 3) transient receptor potential vanilloid receptor 1 (TRPV1R) agonist resiniferatoxin (RTX) (Rudd et al., 2015; Darmani et al., 2020), 4) store-operated Ca^2+^ entry (SOCE) inhibitor MRS 1845 (Li et al., 2013), 5) T-type Ca^2+^ channel blocker Z944 (Casillas-Espinosa et al., 2015), 6) ryanodine receptor (RyR) antagonist dantrolene (Ng et al., 2007), 7) inositol trisphosphate receptor (IP_3_R) antagonist 2-APB (Ng et al., 2007), 8) serotonin type 3 receptor (5-HT_3_R) antagonist palonosetron (Navari, 2013), 9) neurokinin-1 receptor (NK_1_R) antagonist netupitant (Navari, 2013), and 10) dopamine type 2 and 3 receptor (D_2/3_R) antagonist sulpride (Okura et al., 2000).

## Materials and Methods

### Animals

A colony of adult least shrews from the Western University of Health Sciences Animal Facilities were housed in groups of 5–10 on a 14:10 light:dark cycle, and were fed and watered ad libitum. The experimental shrews were 45–60 days old and each weighed 4–6 g. Animal experiments were conducted in accordance with the principles and procedures of the National Institutes of Health Guide for the Care and Use of Laboratory Animals. All protocols were approved by the Institutional Animal Care and Use Committee of Western University of Health Sciences. All efforts were made to minimize animals suffering and to reduce the number of animals used in the experiments.

### Chemicals

The following drugs were used in the present studies: ZD7288, U0126, MRS 1845, Z944 and RTX were purchased from Tocris (Ellisville, MO); ivabradine hydrochloride, nifedipine and sulpride from Sigma/RBI (St. Louis, MO); dantrolene and 2-APB from Santa Cruz Biotechnology (Dallas, TX). Palonosetron and netupitant were kindly provided by Helsinn Health Care (Lugano, Switzerland).

ZD7288, ivabradine hydrochloride and palonosetron were dissolved in water. Nifedipine, U0126, MRS 1845, Z944, dantrolene and 2-APB were dissolved in 25% DMSO in water. Netupitant and RTX were dissolved in a mixture of ethanol/Tween 80/saline at a volume ratio of 1:1:18. Sulpride was dissolved in distilled water with a 10 µL volume of 1/3 concentrated HCl which was then back titrated to pH 5 by the addition of NaOH. All drugs were administered at a volume of 0.1 ml/10 g of body weight.

### Behavioral Emesis Studies

No gender differences between male and female least shrews have been detected in our previous studies. Thus, both males and female shrews were used in the current study. Prior to experimentation, least shrews were brought from the animal facility, separated into individual cages, and were allowed to adapt to the experimental conditions for up to 2 h (h). Daily food was restricted 2 h prior to the start of the experiment, but shrews were given 3–4 mealworms each prior to injection of an emetogen to aid identifying wet vomits as reported previously (Darmani, 1998). For systemic dose-response emesis studies, different groups of shrews were injected with varying doses of ZD7288 (0, 0.05, 0.25, and 1 mg/kg, i. p., *n* = 8 shrews per group). In addition, different groups of shrews (*n* = 6 per group) were injected intracerebroventricularly (i.c.v.) with either vehicle, 2.5, 5 or 10 µg ZD7288. Based on our published stereotaxic atlas of the least shrew brain (Ray and Darmani, 2007), the i. c.v. injection procedure in the least shrew has already been fully described by our laboratory (Darmani et al., 1994; Ray et al., 2009b). Likewise, the emetic effect of a structurally different HCN channel blocker ivabradine (0, 5, and 10 mg/kg, i. p., *n* = 6 per group) was also examined.

Based on the results obtained, i. p. administration of a 1 mg/kg dose of ZD7288 which evoked a maximal frequency of emesis in all tested shrews, was chosen for subsequent behavioral and immunofluorescence staining studies. To evaluate drug interaction studies, different groups of shrews were pretreated with an injection of either corresponding vehicle or varying doses of antiemetics based on our previously published reports: the ERK1/2 inhibitor U0126 (10 and 20 mg/kg, i. p., *n* = 7) (Zhong et al., 2019); LTCC inhibitor nifedipine (1, 2.5, 5 and 10 mg/kg, subcutaneously (s.c.), *n* = 6) (Darmani et al., 2014b); TRPV1R agonist RTX (0.25, 0.5, 1.0 and 2.5 μg/kg, s. c., *n* = 8) (Darmani et al., 2020); SOCE inhibitor MRS 1845 (1, 2.5, 5 and 10 mg/kg, i. p., *n* = 8) (Zhong et al., 2019); T-type Ca^2+^ channel blocker (Z944) (1, 2.5, 5 and 10 mg/kg, i. p., *n* = 9); RyR inhibitor dantrolene (1, 5, and 10 mg/kg, i. p., *n* = 8) (Zhong et al., 2016); IP_3_R inhibitor 2-APB (1, 5, and 10 mg/kg, i. p., *n* = 6) (Zhong et al., 2016); 5-HT_3_R antagonist palonosetron (0.5 mg/kg, s. c, *n* = 6) (Darmani et al., 2014b); NK_1_R antagonist netupitant (5 and 10 mg/kg, i. p., *n* = 6) (Zhong et al., 2019), D_2/3_R antagonist sulpride (8 mg/kg, s. c., *n* = 6) (Darmani et al., 1999). Following 30 min exposure, each pretreated shrew was challenged with a single fully effective emetic-dose of ZD7288 (1 mg/kg, i. p.). Immediately following administrtation of ZD7288, each shrew was placed in the observation cage and the frequency of emesis was recorded for the next 30 min. In the emesis studies the observer was blind to animals’ treatment conditions. Each shrew was used once and then euthanized with isoflurane after the termination of each experiment.

### Immunohistochemistry and Image Analysis

Immunohistochemistry of the least shrew brainstem (20 µm) and jejunal (25 µm) sections was carried out as previously reported (Zhong et al., 2016; Zhong et al., 2018; Zhong et al., 2019; Zhong and Darmani, 2020). The jejunal segment of the least shrew small intestine was dissected in accord with Ray et al. (2009a). The experimenter acquiring and analyzing the images were blind to experimental conditions.

#### Hyperpolarization-Activated Cyclic Nucleotide-Gated Channels Expression

To analyze the expression and distribution of HCN channel subtypes 1–4 in the least shrew brainstem dorsal vagal complex area, the following primary and secondary antibodies were used: rabbit anti-HCN1 antibody (1:300; ab229340, Abcam), mouse anti-HCN2 antibody (1:300; ab84817, Abcam), mouse anti-HCN3 antibody (1:300; ab84818, Abcam), rabbit anti-HCN4 antibody (1:300; APC-052, Alomone Labs), Alexa Fluor 594 donkey anti-rabbit (1:500, Abcam) and 488 conjugated donkey anti-mouse secondary antibodies (1:500, Invitrogen). After washing with PBS, sections were mounted on slides, dried, and cover slipped with antifade mounting medium for staining nuclei with DAPI (Vector Laboratories, H-1500) and. Sections were examined and images (708.5 μm × 708.5 μm) were captured by a confocal microscope (Zeiss LMS 880) at 1024 × 1024 pixels with Zen software using Plan-Apochromat 20×/0.8 M27 objective. Magnified images of each emetic nuclei (AP/NTS/DMNX) of the brainstem dorsal vagal complex were further acquired with Plan-Apochromat 63×/1.4 oil DICM27 objective. This imaging acquisition set-up were applied to the immunohistochemistry performed in this study.

#### c-Fos Expression

To analyze c-Fos expression evoked at 90 min after ZD7288-induced vomiting, c-Fos immunostaining was conducted on both brainstem (20 µm) and jejunal sections (25 µm) from vehicle-treated and ZD7288 (1 mg/kg, i. p.)-treated animals (*n* = 4 shrews per group for brainstem staining; *n* = 3 for jejunual sections staining). Rabbit anti-c-Fos polyclonal antibody (1:5000, ab190289, Abcam) and Alexa Fluor 594 donkey anti-rabbit secondary antibody (1:500, Abcam) were used. The criterion used to differentiate the AP, NTS and DMNX within the DVC has been well recognized (Darmani et al., 2008; Ray et al., 2009a; Chebolu et al., 2010; Zhong et al., 2016; Zhong et al., 2018; Zhong et al., 2019; Zhong and Darmani, 2020). For each animal, c-Fos positive cells in the AP, both sides of NTS and DMNX from 3 sections at 90-μm intervals were counted manually by an experimenter blind to experimental conditions. The average value was used in statistical analysis. Co-staining of jejunum sections with anti-c-Fos antibody and anti-NeuN (neuronal marker) antibody (1:300, MAB377, Millipore) followed by Alexa Fluor 594 donkey anti-rabbit IgG and Alexa Fluor 488 donkey anti-mouse secondary antibodies (1:500, Invitrogen) was conducted to confirm neuronal localization of c-Fos.

#### Co-staining of c-Fos and TPH2

Another set of brainstem sections from vehicle- and ZD7288-treated shrews (*n* = 3 shrews per group) were incubated overnight with primary antibodies (rabbit anti-TPH2 antibody, 1:300, ab111828, Abcam; mouse anti-cFos antibody, 1:1o00, ab208942, Abcam) and incubated with the Alexa Fluor-conjugated secondary antibodies (1:500, Alexa Fluor 488 and 594, Invitrogen). Nuclei of cells were stained with DAPI. The stained sections were examined as described above. For each animal, the number of c-Fos expressing cells among TPH2 positive cells of the selected areas, the AP, both sides of NTS and DMNX, from 3 sections at 90-μm intervals were counted. The mean average value was used for statistical analysis.

#### Phospho-ERK1/2 Immunohistochemistry

To analyze phospho-ERK1/2 expression evoked at 15 min after ZD7288 administration, phospho-ERK1/2 immunofluorescence staining was conducted on brainstem sections from vehicle-treated and ZD7288 (1 mg/kg, i. p.)-treated animals (*n* = 3 shrews per group) with rabbit anti-phospho-ERK1/2 (Thr202/Thr204) (1:500, 4370, Cell Signaling) primary antibody followed by Alexa Fluor 594 donkey anti-rabbit (1:500, Abcam) secondary antibody incubation. Nuclei of cells were stained with DAPI. Integrated density of phospho-ERK1/2 immunoreactivity in the brainstem dorsal vagal complex of 3 sections at 90-μm intervals from each animal of both treatment groups was determined via ImageJ and the mean value per section from individual shrews was used for statistical analysis.

### Statistical Analysis

The frequencies of vomits were analyzed using the Kruskal-Wallis non-parametric one-way analysis of variance (ANOVA) followed by Dunnett’s post hoc test and presented as the mean ± SEM. The percentage of shrews vomiting across treatment groups at different doses was compared using the chi-square test. Statistical significance for differences between two treatment groups was tested by unpaired *t*-test. When more than two treatment groups were compared, a one-way ANOVA was used followed by Dunnett’s post hoc test to determine statistical significance between experimental groups and control. *p* < 0.5 was considered statistically significant.

## Results

### Expression of Hyperpolarization-Activated Cyclic Nucleotide-Gated Channel Subtypes in the Least Shrew Brainstem Dorsal Vagal Complex

**FIGURE 1:**
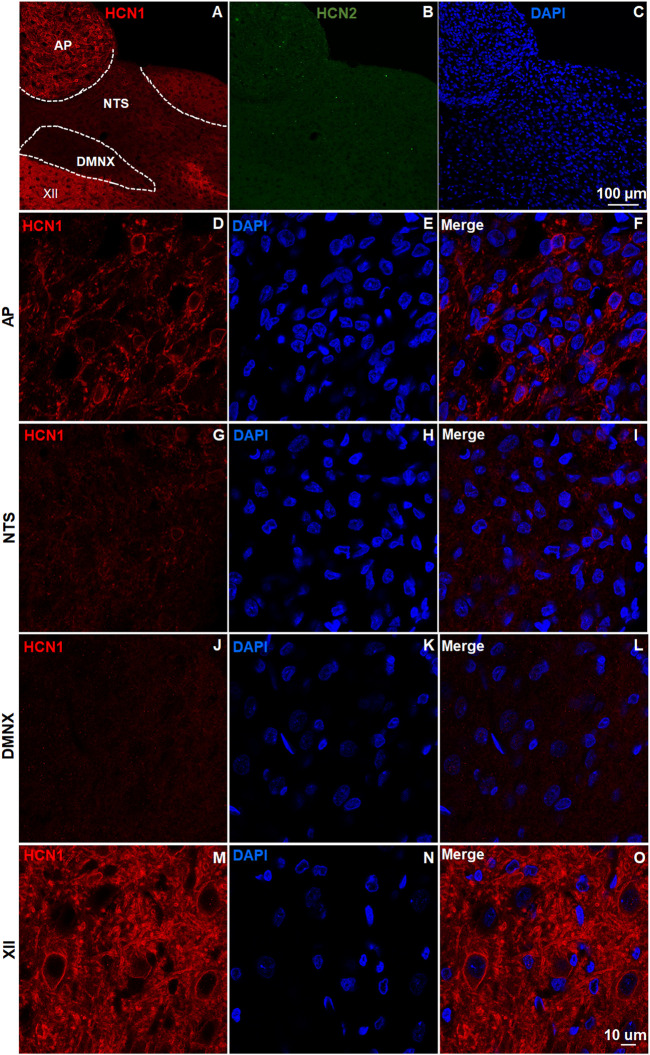
Expression of HCN channel subtypes 1 and 2. HCN channel immunostaining with rabbit anti-HCN1 and mouse anti- HCN2 primary antibodies followed by Alexa Fluor 594 donkey anti-rabbit and 488 donkey anti-mouse secondary antibodies were performed on coronal brainstem sections (20 μm) prepared from naïve least shrews (*n* = 3 shrews). Nuclei were stained with DAPI in blue. **(A-C)** Representative images (20x) show expression of HCN1 but not HCN2 observed in the least shrew brainstem dorsal vagal complex (DVC) area including the three emetic nuclei, the area postrema (AP), the nucleus tractus solitarius (NTS) and the dorsal motor nucleus of the vagus (DMNX). Scale bar, 100 µm. (D–O) Representative images (60x) show differential expression of HCN1 observed in the area postrema (AP), the nucleus tractus solitarius (NTS), the dorsal motor nucleus of the vagus (DMNX) and the hypoglossal nuclei (XII). Scale bar, 50 µm.

**FIGURE 2:**
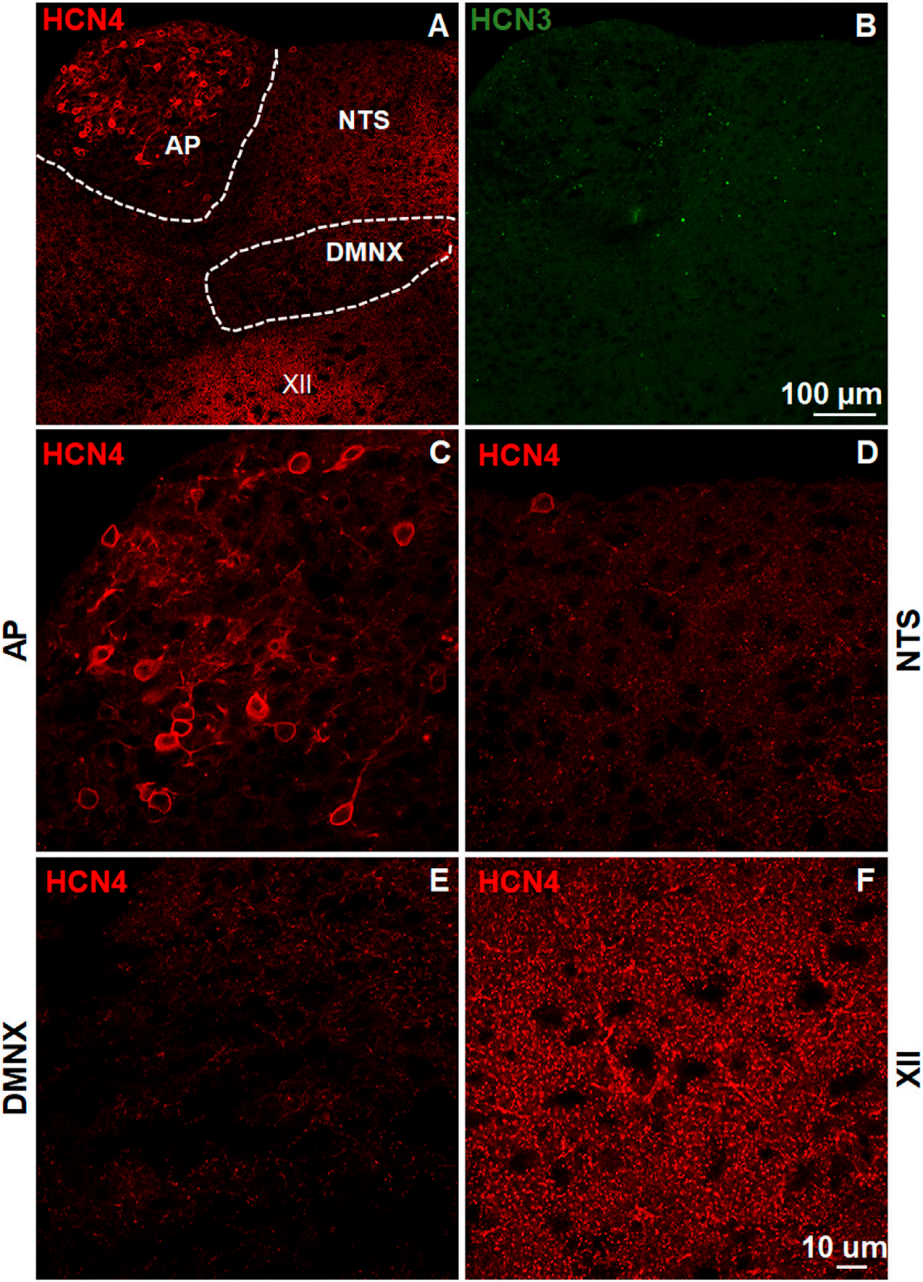
Expression of HCN channel subtypes 3 and 4. HCN channel immunostaining with rabbit anti-HCN4 and mouse anti-HCN3 primary antibodies followed by Alexa Fluor 594 donkey anti-rabbit and 488 donkey anti-mouse secondary antibodies were performed on coronal brainstem sections (20 μm) prepared from naïve least shrews. **(A-B)** Representative images (20x) show expression of HCN4 but not HCN3 observed in the least shrew brainstem dorsal vagal complex (DVC) area including three emetic nuclei, the area postrema (AP), the nucleus tractus solitarius (NTS) and the dorsal motor nucleus of the vagus (DMNX). Scale bar, 100 µm. (C–F) Representative images (60x) show differential expression of HCN4 observed in the area postrema (AP), the nucleus tractus solitarius (NTS), the dorsal motor nucleus of the vagus (DMNX) and the hypoglossal nuclei (XII). Scale bar, 50 µm.

The brainstem DVC constitutes the primary emetic nuclei including the AP, NTS and DMNX. We determined the expression of the HCN channel subtypes (1–4) by immunostaining 20 μm brainstem sections. In the least shrew brainstem DVC area, the most highly expressed HCN channel subtypes were HCN1 (Figure 1) and HCN4 (Figure 2). Both subtypes are highly enriched in the AP as compared with the NTS and DMNX (Figures 1A, 2A). In the AP, both subunits appear to be localized on the membrane and dendrite regions of neurons (Figures 1D, 2C). HCN1 and HCN4 immuno-positive puncta scattered in the NTS (Figures 1G, 2D) and the DMNX (Figures 1J, 2E) at a moderate and low level, respectively. HCN1 and HCN4 were also found to exhibit high level of expression in the hypoglossal nuclei (XII), an area below the DMNX (Figures 1M, 2F). In contrast with the expression pattern of HCN1 and HCN4, expression of HCN2 and HCN3 subtypes were barely visualized in these three nuclei (Figures 1B, 2B).

### ZD7288 Causes Emesis and Activates the Brainstem Emetic Dorsal Vagal Complex Nuclei

**FIGURE 3:**
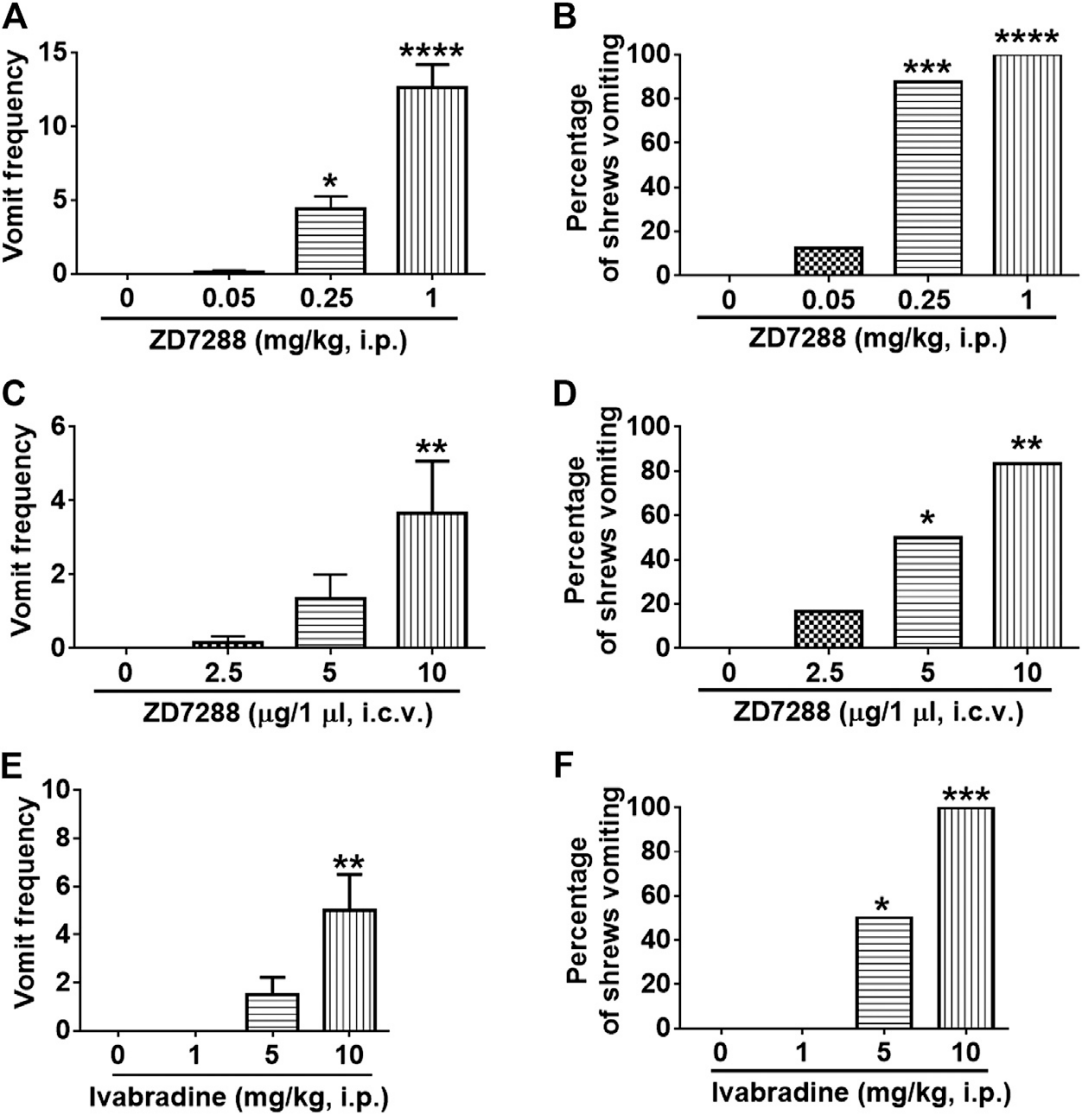
The dose-response emetic effect of the HCN blockers in the lease shrew. Different groups of least shrews were given varying doses of ZD7288 (i.p., *n* = 8 shrews per group or i. c.v., *n* = 6) or ivabradine hydrochloride (i.p., *n* = 6 shrews per group), and were observed for the next 30 min **(A, C, E)** The frequency of emesis was analyzed with Kruskal-Wallis non-parametric one-way ANOVA followed by Dunnett’s post hoc test and presented as mean ± SEM. **(B, D, F)** Percentage of shrews vomiting was analyzed with chi-square test and presented as mean. *p < 0.05, **p < 0.01, ***p < 0.001, ****p < 0.0001 vs. 0 mg/kg.

**FIGURE 4:**
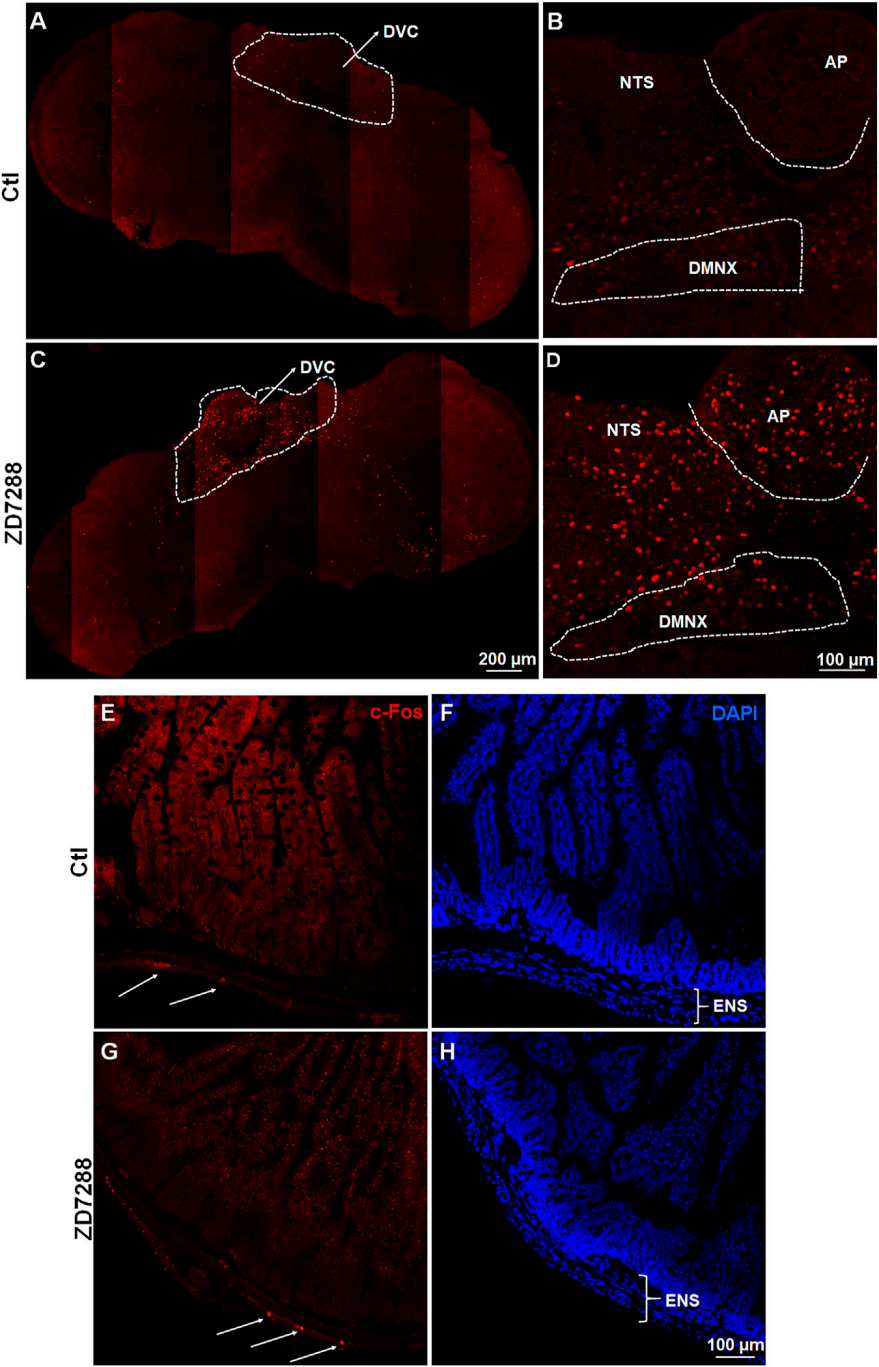
Immunohistochemical analysis of c-Fos following emesis induced by systemic administration of the HCN channel blocker ZD7288. Least shrews were sacrificed 90 min post vehicle treatment, or after the first vomiting occurred post systemic administration (1 mg/kg, i. p.) of ZD7288 (*n* = 4 shrews per group). Shrew brainstem section (20 μm) and intestinal jejunum sections (25 μm) were stained with rabbit c-Fos antibody and Alexa Fluor 594 donkey anti-rabbit secondary antibody. Nuclei were stained with DAPI in blue. **(A, C)** Representative tile-scanned images show a robust c-Fos induction in the brainstem dorsal vagal complex (DVC) in response to ZD7288 (1 mg/kg, i. p.). Scale bar, 100 μm. **(B, D)** Representative single filed images (20x) show c-Fos expression evoked by ZD7288 observed in the DVC emetic nuclei, the area postrema (AP), the nucleus tractus solitarius (NTS) and the dorsal motor nucleus of the vagus (DMNX), within the brainstem. Scale bar, 200 μm. (**E–H**) Representative images (20x) show low c-Fos expression induced by ZD7288 in the enteric nervous system (ENS) of the intestinal jejunum. Scale bar, 100 μm.

**FIGURE 5:**
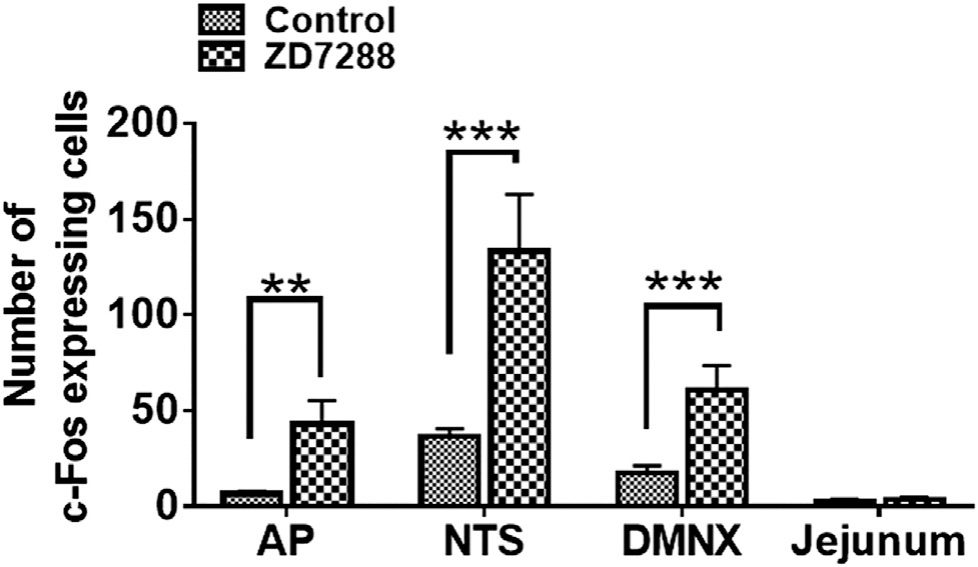
Quantified data for ZD7288-induced c-Fos expression in the least shrew brainstem dorsal vagal complex, containing the area postrema (AP), the nucleus tractus solitarius (NTS) and the dorsal motor nucleus of the vagus (DMNX), as well as the enteric nervous system (ENS) embedded in the wall of jejunum. Values represent the mean number of c-Fos positive cells in each region of interest per section and are presented as mean ± SEM (*n* = 4 shrews per group). *p < 0.05, **p < 0.01, ***p < 0.001 vs. Control (treated with vehicle of ZD7288), Unpaired *t*-test.

**FIGURE 6:**
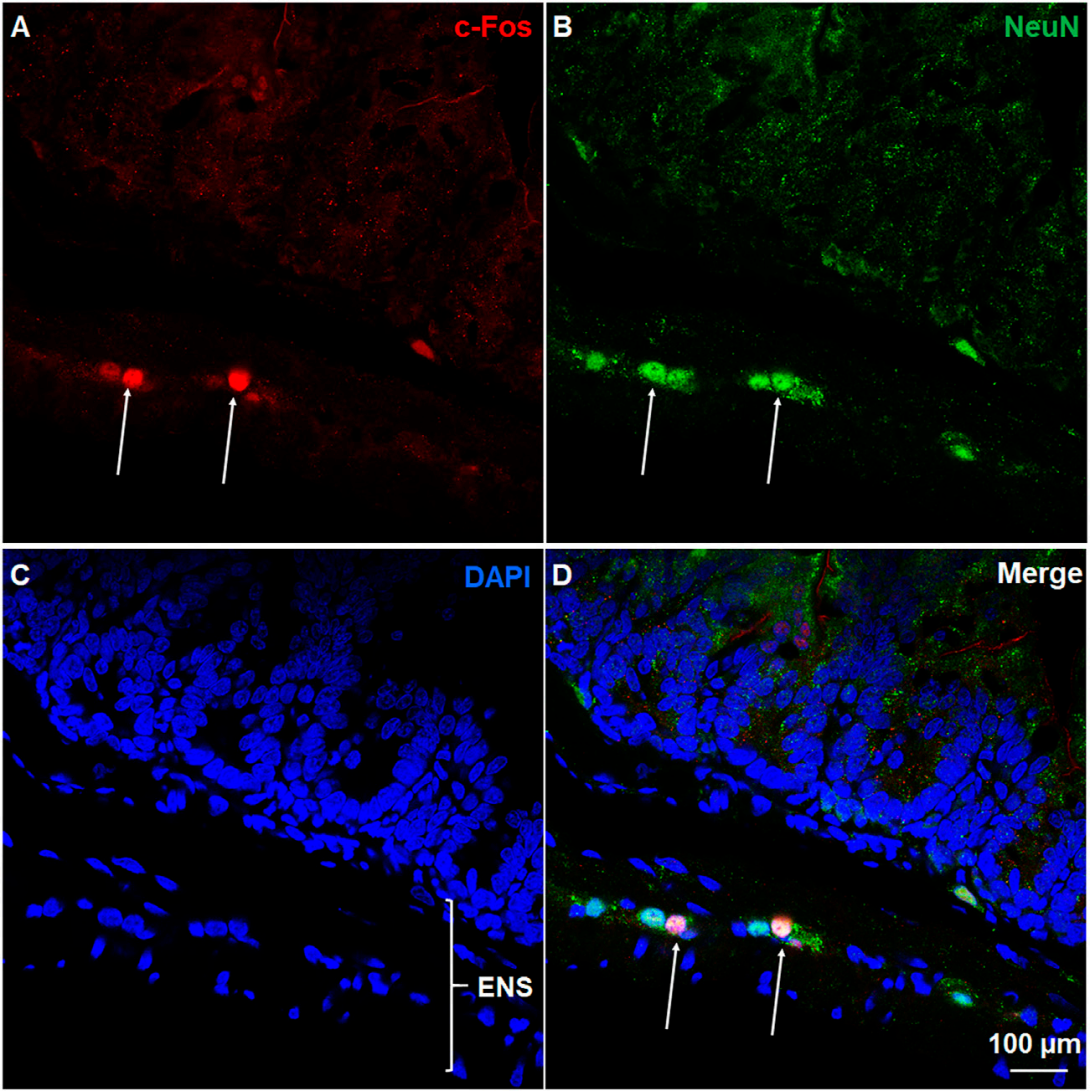
Immunohistochemical analysis of c-Fos expression in NeuN-positive neurons of ENS. Co-staining of jejunum sections from ZD7288 (1 mg/kg, i. p.)-treated shrews with rabbit c-Fos and mouse anti-NeuN antibodies followed by Alexa Fluor 594 donkey anti-rabbit and 488 donkey anti-mouse secondary antibodies. NeuN, a neuronal marker of intrinsic primary afferent neurons of the enteric nervous system. Nuclei were stained with DAPI in blue. **(A–D)** Representative images (60x) show c-Fos expression seen in neurons of the enteric nervous system (ENS), which is embedded in the lining of the intestine. Scale bar, 100 μm.

The dose-response emesis data for ZD7288 are depicted in Figure 3. Intraperitoneal administration of ZD7288 increased the frequency of vomiting in the least shrew in a dose-dependent profile (KW (3, 28) = 27.42, *p* < 0.0001). Dunn’s multiple comparisons post hoc test showed that ZD7288 significantly increased the vomit frequency at its 0.25 (*p* = 0.0364) and 1 mg/kg doses (*p* < 0.0001) (Figure 3A). In addition, the chi-square test indicated that the percentage of animals vomiting in response to ZD7288 also increased dose-dependently (χ^2^ (3, 28) = 25.3, *p* < 0.0001). All shrews vomited at its 1 mg/kg dose (*p* < 0.0001), and 87.5% of shrews vomited at its 0.25 mg/kg (*p* = 0.0004) (Figure 3B). Intracerebroventricular injection of ZD7288 at 0, 2.5, 5 and 10 µg also increased the frequency of emesis in the least shrew in a dose-dependent manner (KW (3, 20) = 11.54, *p* = 0.0091). Dunn’s multiple comparisons post hoc test showed that ZD7288 significantly increased the vomit frequency at its 10 µg dose (*p* = 0.0066) (Figure 3C). In addition, the chi-square test indicated that the percentage of animals exhibiting emesis in response to the centrally administered ZD7288 also increased in a dose-dependent fashion (χ^2^ (3, 20) = 10.49, *p* = 0.0148). Indeed, 83.3% shrews vomited at its 10 µg dose (*p* = 0.0034), and 50% of shrews vomited at its 5 µg (*p* = 0.0455) (Figure 3D). Intraperitoneal injection of HCN channel blocker ivabradine at 0, 1, 5 and 10 mg/kg also increased the frequency of emesis in the least shrew in a dose-dependent manner, with all tested shrews vomited at 10 mg/kg (Figures 3E,F).

c-Fos induction is a recognized tool for evaluation of neuronal activation following peripheral stimulation with an agonist (Bullitt, 1990). Thus, we performed immunohistochemistry to examine c-Fos expression evoked by systemic administration of ZD7288. Relative to the control group treated with vehicle of ZD7288, a 1 mg/kg (i.p.) fully emetic dose of ZD7288 caused marked increases in c-Fos expression in the brainstem DVC throughout the three emetic nuclei, the AP, NTS and DMNX (Figures 4A–D). The numbers of c-Fos-positive cells in each region of interest are delineated in Figure 5. In vehicle-treated shrews, the average values for Fos-positive cells were 6.6 ± 0.6, 36.4 ± 2.1, and 17.2 ± 2 in the AP, NTS, and DMNX, respectively. Following vomiting induced by ZD7288, the average numbers of Fos-positive cells were increased to 43 ± 6.2 in the AP (*p* = 0.0011 vs. Control), 133.3 ± 14.9 in NTS (*p* = 0.0007), and 60.1 ± 6.3 in DMNX (*p* = 0.0006). c-Fos expression in the ENS located in the jejunum was also examined. The ENS demonstrated a low c-Fos staining signal (Figures 4E,G), with the mean number of c-Fos positive cells was 3.556 ± 0.7286 for ZD7288-treated group and 2.111 ± 0.9876 for vehicle-treated group (Figure 5). The statistical analysis showed no significant difference for jejunal c-Fos expression between control and ZD7288-treated groups (*p* = 0.3045) (Figure 5). A further double staining with c-Fos and NeuN antibodies confirmed the jejunal c-Fos expression induced by ZD7288 occurred in NeuN-positive neurons (Figure 6).

**FIGURE 7:**
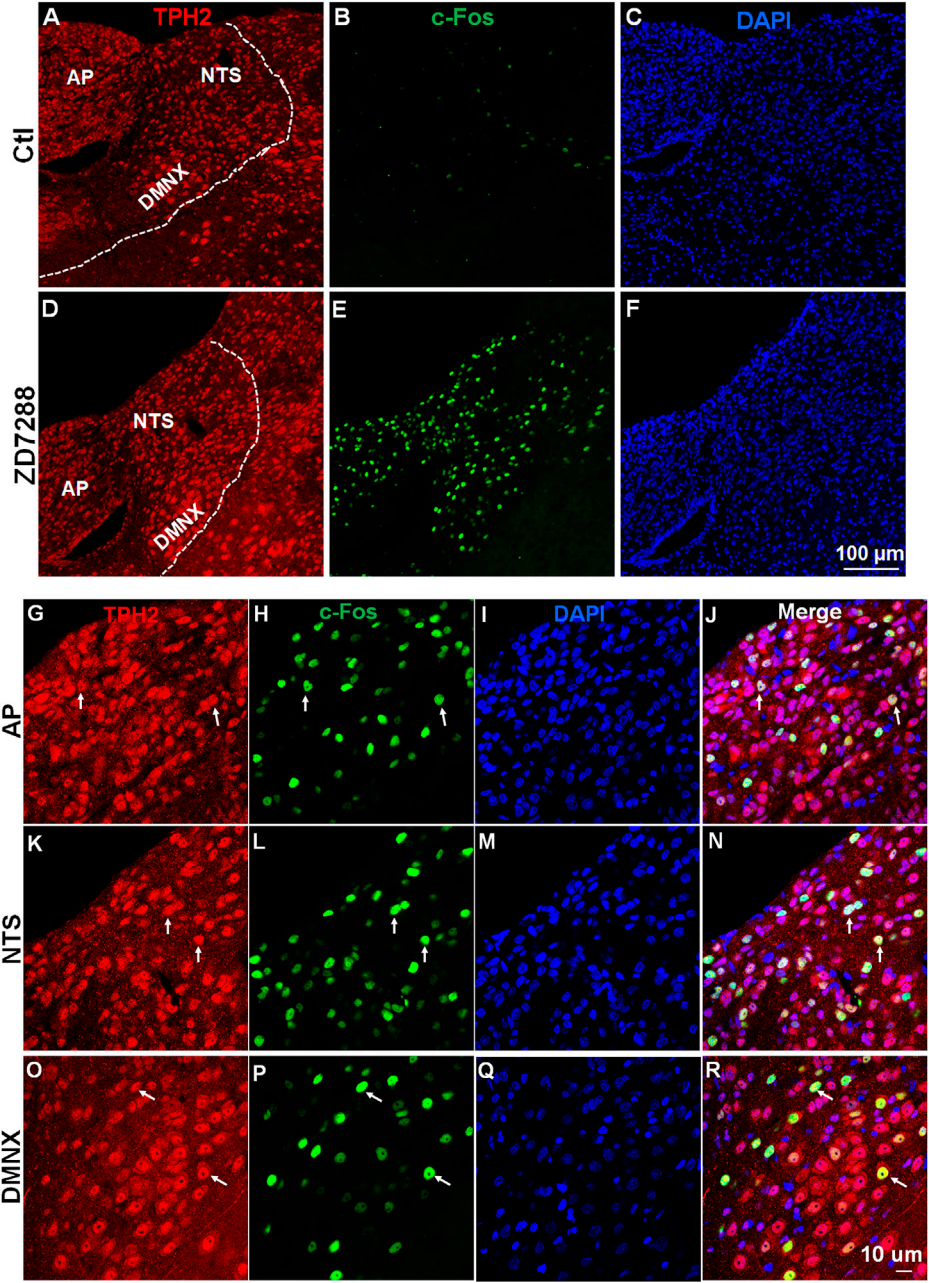
ZD7288 increases c-Fos expression of serotonin neurons in the least shrew brainstem dorsal vagal complex. **(A–F)** Tryptophan hydroxylase 2 (TPH2) is the rate-limiting enzyme in the synthesis of neuronal serotonin. Representative images (20x) showing anti- TPH2, anti-c-Fos and merged immunofluorescence staining in vehicle controls and shrews administered with ZD7288 (1 mg/kg, i. p.) (*n* = 3 shrews per group). Scale bars = 100 μm. (G–R) Representative higher magnification images (×60) showing c-Fos expressing evoked by ZD7288 (1 mg/kg, i. p.) localized in TPH2 positive serotonin neurons of the brainstem dorsal vagal complex, composed of the area postrema (AP), the nucleus tractus solitarius (NTS) and the dorsal motor nucleus of the vagus (DMNX). Scale bars = 10 μm.

A subsequent examination of c-Fos expression in TPH2-positive serotonergic neurons by double staining brainstem sections with c-Fos and TPH2 antibodies established that activation of serotonergic neurons in the brainstem DVC, composed of the AP, NTS and DMNX, induced by ZD7288 (Figure 7). As shown in Supplementary Figure S1, in vehicle-treated shrews, the average values for c-Fos expressing TPH2 positive cells were 5.2 ± 0.3, 37.6 ± 6.3, and 12.1 ± 1.4 in the AP, NTS, and DMNX, respectively. Following vomiting induced by ZD7288, the average numbers of c-Fos expression in TPH2-positive cells were increased to 52.6 ± 7.8 in the AP (*p* = 0.0037 vs. Control), 133.1 ± 9.2 in NTS (*p* = 0.001), and 61.9 ± 4.9 in DMNX (*p* = 0.0006).

### Emesis Induced by ZD7288 Involves ERK1/2 Phosphorylation

**FIGURE 8:**
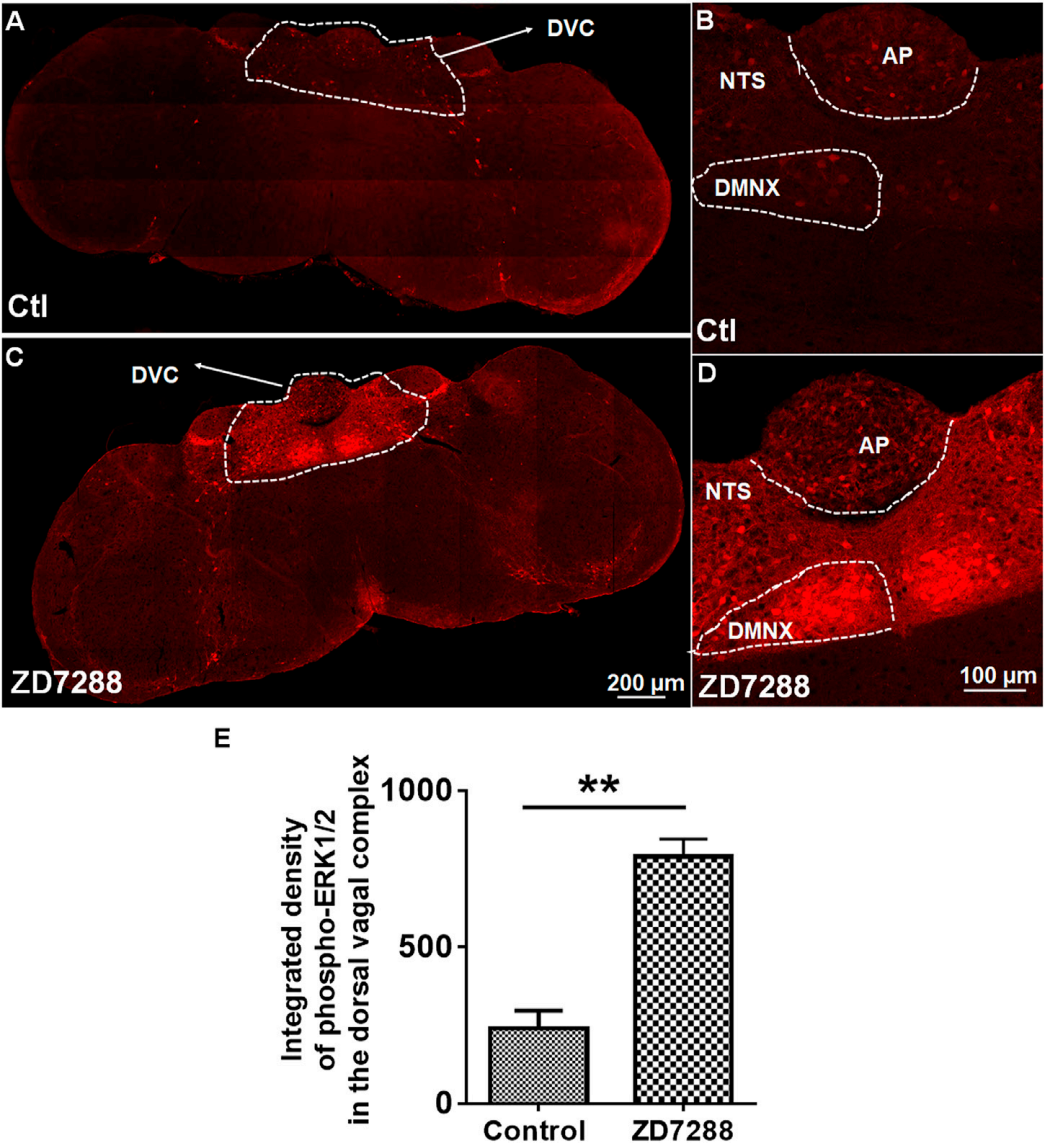
Immunohistochemical analysis of ERK1/2 phosphorylation following the HCN channel blocker ZD7288-induced emesis. Least shrews were sacrificed 15 min after vehicle or ZD7288 injection (1 mg/kg, i. p.) (*n* = 3 shrews per group). Brainstem sections (20 μm) and intestinal jejunum sections (25 μm) were stained with rabbit anti-phospho-ERK1/2 antibody and Alexa Fluor 594 donkey anti-rabbit secondary antibody. Nuclei were stained with DAPI in blue. **(A, C)** Representative tile scan images show a strong upregulation of ERK1/2 phosphorylation (pERK) in the brainstem dorsal vagal complex (DVC) in response to ZD7288. Scale bar, 200 μm. **(B**, **D)** Representative images show ERK1/2 phosphorylation evoked by ZD7288 seen in the DVC throughout three emetic nuclei, the area postrema (AP), the nucleus tractus solitarius (NTS) and the dorsal motor nucleus of the vagus (DMNX). Scale bar, 100 μm. **(E)** Statistical analysis of integrated density of ERK1/2 phosphorylation evoked by ZD7288 (1 mg/kg, i. p.) in the brainstem dorsal vagal complex. **p < 0.01 vs. Control, Unpaired *t*-test.

To determine the participation of ERK1/2 in ZD7288-induced emesis, immunohistochemistry was performed to examine ERK1/2 phosphorylation evoked by ZD7288 (1 mg/kg, i. p.). Representative images from tile scanning of stained sections show that 15 min post ZD7288 administration, a significant increase in phosphorylation of ERK1/2 occurred in the brainstem DVC (Figures 8A,C). Single field images acquired under ×20 objective show ZD7288 evoked significant ERK1/2 phosphorylation (*p* = 0.0024) in brainstem dorsal vagal complex (Figures 8B,D,E).

**FIGURE 9:**
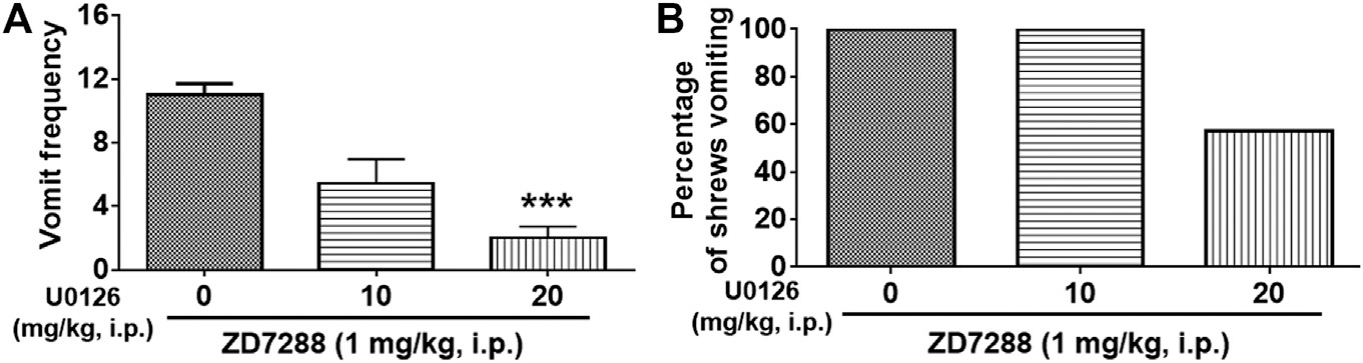
Effect of ERK1/2 inhibitor U0126 on the HCN channel blocker ZD7288-induced emesis. Different groups of shrews were given vehicle or varying doses of the ERK1/2 inhibitor U0126 (i.p.) (*n* = 7 shrews per group), 30 min prior to ZD7288 (1 mg/kg, i. p.) administration. Shrews were observed the next 30 min. **(A)** The frequency of emesis was analyzed with Kruskal-Wallis non-parametric one-way ANOVA followed by Dunnett’s post hoc test and presented as mean ± SEM. **(B)** Percentage of shrews vomiting was analyzed with chi-square test and presented as mean. ***p < 0.001 vs. 0 mg/kg.

Next, we evaluated the antiemetic potential of ERK1/2 inhibitor U0126 behaviorally (Figure 9). Different groups of shrews were pretreated with U0126 (0, 10 and 20 mg/kg, i. p.) 30 min prior to ZD7288 (1 mg/kg, i. p.) injection. As displayed in Figures 9A,B, U0126 pretreatment attenuated the frequency of ZD7288-induced vomiting (KW (2, 18) = 12.55, *p* = 0.0003), with a significant reduction in the vomit frequency observed at its 20 mg/kg dose (*p* = 0.0008) (Figure 9A). The chi-square test showed U0126 pretreatment tended to reduce the percentage of shrews vomiting (χ^2^ (2, 18) = 7.2, *p* = 0.0302) in response to ZD7288, but failed to achieve significance at the 20 mg/kg dose (*p* = 0.0507) (Figure 9B).

### Ca^2+^ Channel Modulators at the Cell Membrane Reduce ZD7288-Induced Emesis

#### Antiemetic Effect of L-Type Ca^2+^ Channel Inhibitor

**FIGURE 10:**
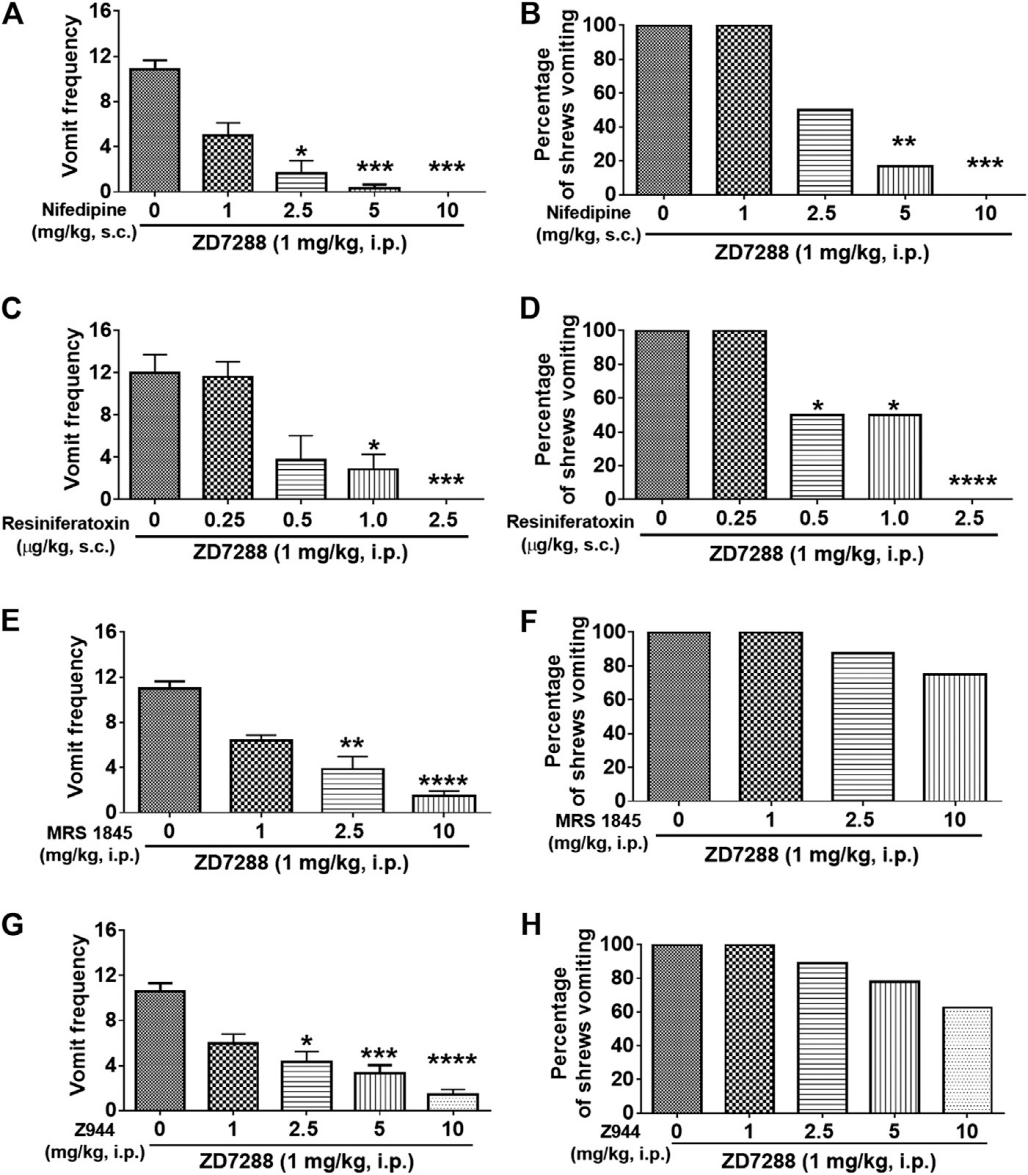
Effects of modulators of cell membrane Ca^2+^ channels on the HCN channel blocker ZD7288-induced emesis. Different groups of least shrews were given an injection of either the corresponding vehicle, or varying doses of: 1) The L-type Ca^2+^ channel (LTCC) inhibitor nifedipine (s.c.) (*n* = 6 shews per group); 2) the TRPV1R agonist resiniferatoxin (RTX) (s.c.) (*n* = 8); 3) store-operated Ca^2+^ entry blocker MRS 1845 (i.p.) (*n* = 8); 4) T-type Ca^2+^ channel inhibitor Z944 (i.p.) (*n* = 9), 30 min prior to ZD7288 injection (1 mg/kg, i. p.). Emetic parameters were recorded for the next 30 min **(A, C, E, G)** The frequency of emesis was analyzed with Kruskal-Wallis non-parametric one-way ANOVA followed by Dunnett’s post hoc test and presented as mean ± SEM. **(B, D, F, H)** Percentage of shrews vomiting was analyzed with chi-square test and presented as mean. *p < 0.05, **p < 0.01, ***p < 0.001, ****p < 0.0001 vs. 0 mg/kg.

Dose-dependent broad-spectrum antiemetic efficacy of the LTCC inhibitor nifedipine has previously been demonstrated in the least shrew model of emesis in our laboratory (Darmani et al., 2014b; Zhong et al., 2016). In this study, pretreatment with nifedipine (1, 2.5, 5 and 10 mg/kg, s. c.) 30 min before ZD7288 (1 mg/kg, i. p.) administration, caused a dose-dependent suppression of both mean vomit frequency [KW (4, 25) = 23.98, *p* < 0.0001] and the percentage of shrews vomiting [χ^2^ (3, 37) = 20.63; *p* = 0.0004] in response to ZD7288 administration (Figure 10). Indeed, relative to the control group pretreated with vehicle of nifedipine, nifedipine (2.5 and 5 mg/kg, s. c.) induced substantial decreases in both the mean vomit frequency in response to ZD7288 (*p* = 0.0104 and *p* = 0.0006, respectively) (Figure 10A), as well as the number of shrews vomiting (*p* = 0.1818 and *p* = 0.0034, respectively) (Figure 10B). Moreover, nifedipine at 10 mg/kg completely blocked ZD7288-evoked vomiting (*p* = 0.0001 for frequency and *p* = 0.0005 for percentage).

#### Anti-Emetic Effect of the TRPV1 Receptor Agonist

The TRPV1 agonist, resiniferatoxin (RTX) at low nanomolar nonemetic doses has the capacity to completely block vomiting caused by diverse emetogens in least shrews (Darmani et al., 2020). Thus, the antiemetic efficacy of RTX was next tested against ZD7288 (1 mg/kg, i. p.)-induced vomiting. Significant reductions in a dose-dependent fashion were observed in both the mean vomit frequency [KW (4, 34) = 24.81, *p* < 0.0001] (Figure 10C) and percentage of shrews vomiting [χ^2^ (4, 34) = 23.33; *p* = 0.0001] (Figure 10D). A significant reduction in the mean vomit frequency occurred from 1.0 μg/kg dose, whereas significance in the percentage of shrews vomiting began from its 0.5 μg/kg dose. Moreover, complete suppression of both emetic parameters occurred at 2.5 μg/kg dose of RTX (*p* = 0.0005 and *p* < 0.0001, respectively) (Figures 10C,D).

#### Antiemetic Effect of SOCE Inhibitor

Store-operated Ca^2+^ entry (SOCE) is an important route by which Ca^2+^ mobilization occurs (Mizuta et al., 2008). As shown in Figures 10E,F, the SOCE inhibitor MRS 1845 dose-dependently attenuated the frequency of ZD7288-induced vomiting [KW (3, 28) = 24.81, *p* < 0.0001], with significant attenuation observed at its 2.5 (*p* = 0.0023) and 10 mg/kg doses (*p* < 0.0001). However, MRS 1845 had no significant effect on the percentage of shrews vomiting [χ^2^ (3, 28) = 4.046; *p* = 0.2565].

#### Antiemetic Effect of T-type Ca^2+^ Channel Inhibitor

As shown in Figures 10G,H, the selective T-type Ca^2+^ channel inhibitor Z944 dose-dependently attenuated the frequency of ZD7288-induced vomiting [KW (4, 40) = 29.21, *p* < 0.0001], with significant decrease observed at its 2.5 (*p* = 0.0113), 5 (*p* = 0.0009) and 10 mg/kg doses (*p* < 0.0001), but had no significant impact on the percentage of shrews vomiting [χ^2^ (4, 40) = 6.538; *p* = 0.1624].

### Intracellular Ca^2+^ Channel Modulators Reduce ZD7288-Evoked Emesis

**FIGURE 11:**
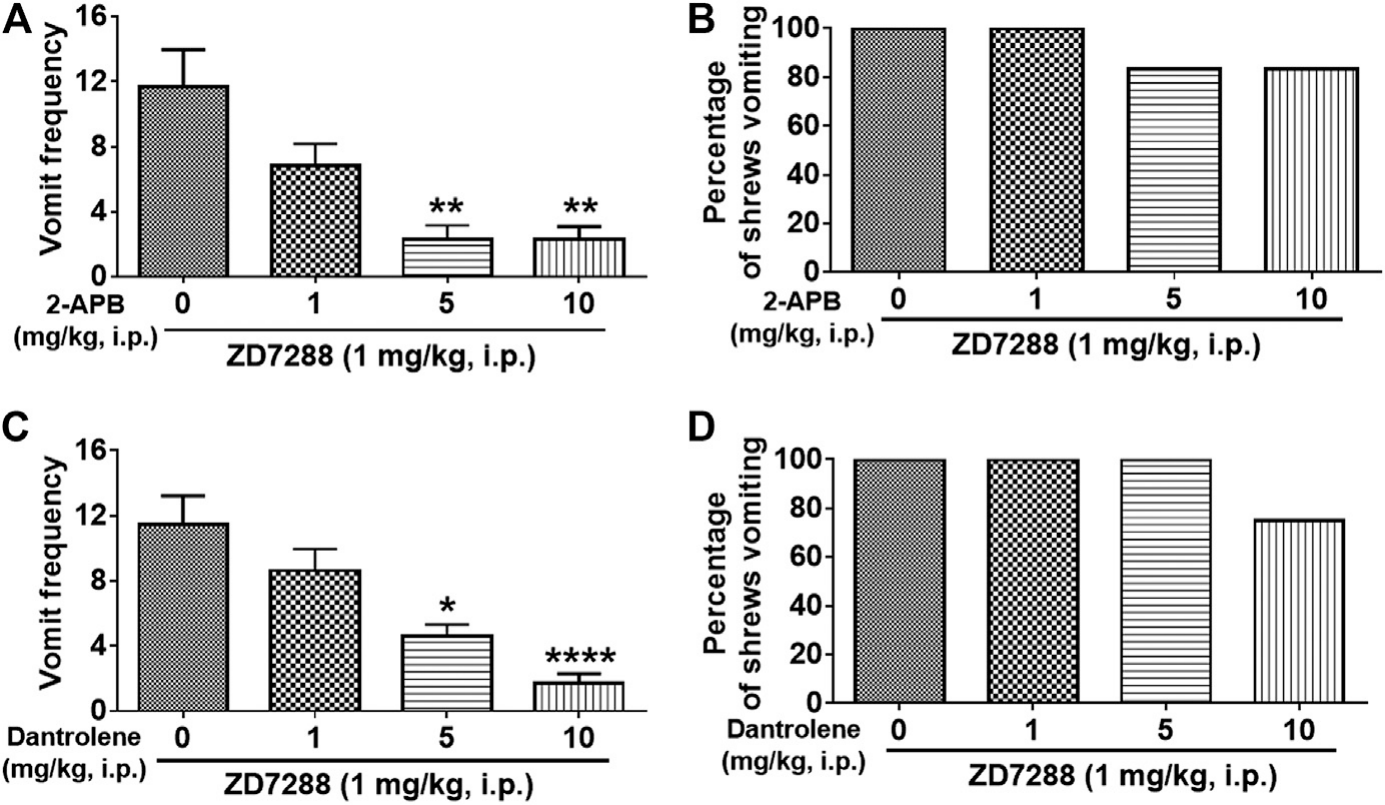
Effects of intracellular Ca^2+^ channel modulators on the HCN channel blocker ZD7288-induced emesis. Thirty minutes prior to an injection of ZD7288 (1 mg/kg, i. p.), different groups of least shrews were given an injection (i.p.) of either the corresponding vehicle, or varying doses of: 1) the ryanodine receptor (RyR) antagonist dantrolene (*n* = 8 shrews per group) **(A, B)**, and 2) the inositol-1, 4, 5-triphosphate receptor IP_3_R antagonist 2-APB (*n* = 6) **(C, D)**. Emetic parameters were recorded for the next 30 min post ZD8288 injection. **(A, C)** The frequency of emesis was analyzed with Kruskal-Wallis non-parametric one-way ANOVA followed by Dunnett’s post hoc test and presented as mean ± SEM. **(B, D)** Percentage of shrews vomiting was analyzed with chi-square test and presented as mean. *p < 0.05, **p < 0.01, ****p < 0.0001 vs. 0 mg/kg.

We next investigated whether intracellular Ca^2+^ release channels such as inositol trisphosphate receptors (IP_3_Rs) and/or ryanodine receptors (RyRs), are involved in ZD7288-induced vomiting. The IP_3_R inhibitor 2-APB (1, 5, and 10 mg/kg) attenuated ZD7288-evoked vomiting frequency [KW (3, 20) = 15.08, *p* = 0.0017] with significant reductions occurring at its 5 (*p* = 0.0038) and 10 mg/kg (*p* = 0.0041) doses (Figure 11A). However, none of the 2-APB tested doses significantly affected the percentage of shrews vomiting [χ^2^ (3, 20) = 2.182, *p* = 0.5355] (Figure 11B). Figure 11C demonstrates that pretreatment with the RyR inhibitor dantrolene (0.1, 0.5, 1, 2.5 and 5 mg/kg, i. p.), significantly and in a dose-dependent manner suppressed ZD7288-evoked vomit frequency [KW (3, 28) = 21.36, *p* < 0.0001] with significant reductions observed at its 5 (*p* = 0.0231) and 10 mg/kg doses (*p* < 0.0001). However, dantrolene also failed to significantly reduce the percentage of shrews vomiting in response to ZD7288 [χ^2^ (3, 28) = 6.4, *p* = 0.0937] (Figure 11D).

### Receptor Antagonists Reduce ZD7288-Evoked Emesis

**FIGURE 12:**
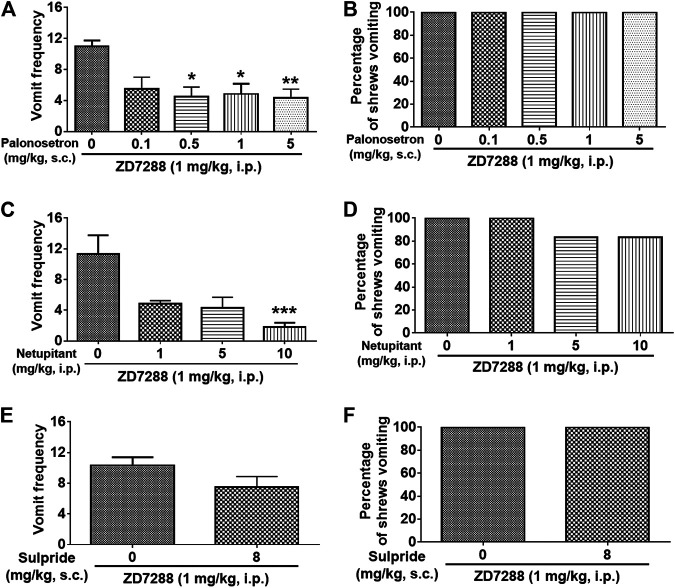
Efficacy of receptor-selective antiemetics against the HCN channel blocker ZD7288-induced emesis. Different groups of least shrews were given an injection of either the corresponding vehicles (0 mg/kg), or varying doses of 5-HT_3_R antagonist palonosetron (s.c.) (*n* = 6 shrews per group) (A and B), NK_1_R antagonist netupitant (i.p.) (*n* = 6) (C and D), or the D_2/3_R antagonist sulpride (s.c.) (*n* = 6) (E and F), 30 min prior to ZD7288 administration (1 mg/kg, i. p.). Emetic parameters were recorded for the next 30 min **(A, C)** The frequency of emesis was analyzed with Kruskal-Wallis non-parametric one-way ANOVA followed by Dunnett’s post hoc test and presented as mean ± SEM. **(E)** The frequency of emesis was analyzed with Unpaired *t*-test and presented as mean ± SEM. **(B, D, F)** Percentage of shrews vomiting was analyzed with chi-square test and presented as mean. *p < 0.05, **p < 0.01, ***p < 0.001 vs. 0 mg/kg.

To evaluate a role for serotonergic 5-HT_3_, neurokinin NK_1_ receptors and dopaminergic D_2_ receptors in ZD7288-induced vomiting, different groups of shrews were pretreated with either the 5-HT_3_R antagonist palonosetron (0.1, 0.5, 1 and 5 mg/kg, s. c.), the NK_1_R antagonist netupitant (1, 5 and 10 mg/kg, i. p.), or the D_2_R antagonist sulpride (8 mg/kg, i. p.) for 30 min prior to ZD7288 (1 mg/kg, i. p.) injection. Palonosetron pretreatment reduced the mean vomit frequency [KW (4, 25) = 13, *p* = 0.0113] in response to ZD7288 (Figure 12A), with significant reduction occurring at its 0.5 (*p* = 0.0134), 1 (*p* = 0.0204) and 5 (*p* = 0.0092) doses. The chi-square test indicated that palonosetron failed to protect shrews from vomiting (Figure 12B). Netupitant (1, 5 and 10 mg/kg, i. p.) also caused a dose-dependent decrease in the frequency of evoked vomits [(KW (3, 20) = 14.91, *p* = 0.0019)] with a significant reduction seen at its 10 (*p* = 0.0004) (Figure 12C). The chi-square test indicates that the number of shrews vomiting in response to ZD7288 was not significantly affected by netupitant [(χ^2^ (3, 20) = 2.182, *p* = 0.5355)] (Figure 12D). Sulpride at 8 mg/kg had no effects on the mean vomit frequency (*p* = 0.1345) and the percentage of shrews vomiting (*p* > 0.9999) in response to ZD7288 (Figures 12E,F).

## Discussion

For the first time this study demonstrates that the HCN channel blocker ZD7288 evokes robust emetic behavior in least shrews when administered either centrally or peripherally. The emetic process is accompanied by activation of serotoninergic neurons in the brainstem emetic loci and is sensitive to Ca^2+^ channel modulators as well as other emetic receptor antagonists.

### Multifaceted Functions of Hyperpolarization-Activated Cyclic Nucleotide-Gated Channels

HCN channels are expressed in the heart and are known to have a critical role in controlling cardiac pacemaker activity (Rivolta et al., 2020). Ivabradine is the first clinically approved drug to efficiently treat heart failure (Postea and Biel, 2011) and cancer chemotherapy-evoked left ventricular dysfunction (Sarocchi et al., 2018). In agreement with clinical findings (Chaturvedi et al., 2013), in this study administration of ivabradine caused dose-dependent vomiting in all tested least shrews at 10 mg/kg (i.p.). In the nervous system, HCN channels are widely expressed in central and peripheral neurons where they play important roles in regulating excitability and neuronal firing (McGovern et al., 2014). They are known to be involved in epilepsies and neuropathic pain disorders, both of which are characterized by enhanced neuronal firing patterns (Postea and Biel, 2011). In addition, peripheral neuropathy evoked by oxaliplatin, a platinum-based chemotherapeutic agent, has been linked to activation of HCN channels-mediated currents (*I*_h_), and ivabradine can attenuate this effect (Chang et al., 2020). Thus, HCN channels are considered as promising targets for anticonvulsant and analgesic drug discovery (Postea and Biel, 2011). Indeed, the HCN channel blocker ZD7288 can inhibit hippocampal synaptic plasticity in rats (Zhang et al., 2016) and reduces the generation of hippocampal epileptic discharges in rabbits (Kitayama et al., 2003). Furthermore, systemic, or local administration of ZD7288 suppresses neuropathic pain behavior as well as abnormal spontaneous firing in injured nerve fibers in rats, supporting the accumulating evidence that increased HCN channel activity is responsible for neuropathic pain (Chaplan et al., 2003; Lee et al., 2005; Luo et al., 2007; Ding et al., 2018). Moreover, HCN channel inhibition with ZD7288 provides protection for neural stem cells during radiotherapy, suggesting new therapeutic strategies against neurocognitive damage caused by chemotherapy and radiotherapy in cancer patients (Johard et al., 2020).

The area postrema lacks a complete blood-brain-barrier and some circulating substances can easily enter the brainstem (Borison, 1989; Miller and Leslie, 1994). HCN channel activation in the area postrema (Milligan et al., 2006) should be important for emetic neuronal activity since a reduction in HCN channel-mediated current is associated with decreased neuronal excitability (Kitayama et al., 2003; Zhang et al., 2016). Thus, antiemetic effects should be expected when neurons in the area postrema are exposed to HCN channel blockers such as ZD7288 (Funahashi et al., 2003) or ivabradine (Funahashi et al., 2004). However, our study demonstrates the emetic effects of these two structurally different HCN blockers when administered systemically. Although ZD7288 can inhibit T-type Ca^2+^ channels activity (Felix et al., 2003), the possibility that ZD7288 may exert an emetic effect through blocking T-type Ca^2+^ channel can be excluded in this study. Indeed, ZD7288 is shown to be 20 times more selective for HCN channel than for T-type Ca^2+^ channels (Sánchez-Alonso et al., 2008), and the selective and potent T-type Ca^2+^ channel blocker Z944 (Casillas-Espinosa et al., 2015) lacks emetic activity in least shrews when administered intraperitoneally (data not shown). The current study is an extension of a published study in a vomit incompetent species (conditioned taste aversion test in rats), which had suggested a possible antiemetic/antinausea effect of ZD7288 at low doses vs. its probable pro-emetic potential at doses larger than 1 mg/kg (Shinpo et al., 2012). However, since ivabradine can evoke emesis in vomit competent species such least shrews and in angina patients at low doses (Chaturvedi et al., 2013), our notion that these structurally different HCN channel blockers exert emetic activity via blockade of HCN channels is a better probability (Figure 3). In fact, both upregulation and downregulation of HCN channels have been associated with increased neuronal excitability (Doan et al., 2004; Postea and Biel, 2011).

### Translational Significance

In this preclinical setting our study demonstrates the potential side-effects of HCN channel inhibitors/blockers in the process of clinical drug discovery. HCN channels are overexpressed in inflammatory and neuropathic pain states (Ramírez et al., 2018). In fact, HCN blockers reduce neuronal excitability and pain perception in animal studies and HCN channels are considered as pharmacological targets for pain management in patients (Ramírez et al., 2018). The HCN channel blocker used in the present study, ZD7288, is a widely used pharmacological tool to study both the function of these channels as well as its analgesic effect in various rodent pain models (Herrmann et al., 2015; Ramírez et al., 2018). Additional studies also demonstrate pain-attenuating efficacy of other HCN blockers including ivabradine and compound 12 m (Ramírez et al., 2018; He et al., 2019). The current study demonstrates that not only systemic (i.p.) administration of ZD7288 is proemetic in a dose-dependent manner, but other HCN blockers such as ivabradine and its analog cilobradine which ate are structurally different from ZD7288 (Bucchi et al., 2013), also evoke vomiting in least shrews. Furthermore, HCN channels have been implicated in the pathogenesis of epilepsy and the efficacy of HCN blockers used as antiseizure drugs in animal studies have recently been reviewed (Kharouf et al., 2020a; Kharouf et al., 2020b). Thus, in the above examples it seems quite clear that the emetic potential of new HCN blockers should be investigated in vomit-competent species prior to further clinical development. In fact, such a case already exists in the clinical development of phosphodiesterase inhibitors (Peng et al., 2020).

Our study provides the first evidence for involvement of HCN channels in the mediation of emesis in a laboratory animal model of vomiting. In fact, HCN channel blockers ivabradine and cilobradine are equipotent on HCN4 and HCN1 channels, while ZD7288 is more selective for HCN1 over HCN4 (Novella Romanelli et al., 2016). In the current study, ZD7288 at 1 mg/kg dose was a fully effective emetogen in all tested shrews. Ivabradine and cilobradine (DK-AH 269) also induced vomiting in all tested least shrews, but only at the 10 mg/kg dose. Thus, our findings not only demonstrate that HCN1 may be involved in vomiting induced by ZD7288, but also shows efficacy differences in the capacity of HCN blockers in evoking emesis. Of further interest, there are several well-known proemetic drugs with additional HCN channel-blocking activities:

(1) Nicotine is a cholinergic agonist with proemetic effects in animals when given subcutaneously (Beleslin et al., 1981; Ueno et al., 1987; Yamamoto et al., 2004; Parker et al., 2009; Horn et al., 2014). Moreover, neuronal excitability induced by nicotine has been shown to be mediated via binding and inhibiting the HCN channels (Griguoli et al., 2010; Kodirov et al., 2014).
(2) Loperamide, an opiate receptor agonist, commonly used in the treatment of diarrhea, reliably induces emesis in the ferret when given subcutaneously (Bhandari et al., 1992; Zaman et al., 2000). Moreover, loperamide can block HCN1 more potently than HCN4 (Lee et al., 2008).
(3) The inhalation anesthesthetic isoflurane causes emesis in the musk shrews (Horn et al., 2014). Isoflurane has been shown to inhibit the activity of HCN1 subunit containing HCN channels and increased the cortical neuronal excitability (Chen et al., 2009). Other anesthetics such as lidocaine and its metabolite, monoethylglycinexylidide, possess inhibitory actions on HCN channels (Meng et al., 2011).

Therefore, the HCN channel subunit 1 (HCN1) may potentially represent an important mediator in nausea and vomiting.

### ZD7288 Evokes Vomiting Through Central Activation of Serotonin Neurons

The expression pattern of HCN subtypes (1–4) vary in the central nervous system and at different levels in the brainstem (Shah, 2014). Furthermore, HCN1 and HCN3 channels were suggested to be the predominant forms in area postrema neurons (Monteggia et al., 2000), which is inconsistent with our present immunohistochemical staining showing besides HCN1, strong HCN4 immunoreactivity in the AP. Moreover, our study more specifically demonstrates their differential distribution among the DVC emetic nuclei of the brainstem. Although it remains unknown whether ZD7288 can cross the blood-brain barrier, circulating ZD7288 following its i. p. injection can act centrally on the brainstem area postrema which lacks a blood-brain barrier (Borison, 1989; Miller and Leslie, 1994). In fact, systemic administration of small doses of ZD7288 (e.g., 1 mg/kg) evokes vomiting in all tested shrews which is accompanied by significant c-Fos expression in the brainstem emetic nuclei, including the AP, NTS and DMNX. On the other hand, i. p.-administered ZD7288 only caused minimal c-Fos expression in a small number of neurons of the jejunal ENS, suggesting central emetic sites may play the principal role in ZD7288-induced emesis. The high levels of HCN1 and HCN4 in the AP area also support the notion of a central emetic site of action of bloodborne ZD7288 acting directly on HCN channels of the AP area. Although neurons residing in the ENS modulate intestinal motility and secretion (Furness, 2000; Sasselli et al., 2012), it appears that the peripheral emetic loci play a limited role in ZD7288-induced vomiting.

This above unlike the case of our previous studies where pronounced immunoreactivity in both c-Fos and ERK1/2 phosphorylation were observed in the jejunal ENS following emesis evoked by the Akt inhibitor MK-2206 (10 mg/kg, i. p.), suggesting the involvement of peripheral emetic loci in MK-2206-evoked emesis (Zhong et al., 2021). The jejunal ENS also exhibits significant c-Fos expression (relative to vehicle injection) following emesis induced by chemotherapeutic agent cisplatin as well as receptor selective emetogens, such as the serotonin 5-HT_3_ receptor agonist 2-Methyl-5-HT, dopamine D_2_R agonist quinpirole and neurokinin NK_1_R agonist GR73632 (Ray et al., 2009a). Therefore, in this study, the strong c-Fos and phospho-ERK1/2 upregulation observed in the brainstem DVC, but not in the ENS in response to systemically administered ZD7288 could be considered as an evidence for central emetic action of ZD7288. The CNS neurons containing TPH2, the rate-limiting enzyme in 5-HT synthesis, are regarded as serotonergic cells (Cai et al., 2014). In the present study, it is noteworthy that following ZD7288 administration at its fully effective emetic dosage, c-Fos expression was exclusively increased in TPH2-expressing serotoninergic neurons of the dorsal vagal complex, indicating that circulating ZD7288 directly activate serotonergic neurons in the dorsal vagal complex, which probably leads to 5-HT release which would subsequently stimulate 5-HT_3_ receptors to induce vomiting.

Both c-Fos and pERK1/2 have been applied as neuronal activation indicators *in vivo* (Ferrini et al., 2014). Our published findings implicate ERK1/2 phosphorylation in the brainstem as one common event in the emetic responses elicited by i. p.-injection of distinct emetogens including the: 1) substance P neurokinin NK_1_ receptor agonist GR73632, 2) LTCC activator FPL64176, 3) intracellular Ca^2+^ signaling amplifier thapsigargin, 4) the serotonin 5-HT_3_ receptor agonist 2-Methyl-5-HT (Zhong et al., 2014; Zhong et al., 2016; Zhong et al., 2018; Zhong et al., 2019), and 5) chemotherapeutic agent cisplatin (Darmani et al., 2015). Consistent with these findings, in the present study we find significant phosphorylation of ERK1/2 in the DVC emetic nuclei (AP, NTS and DMNX) within the least shrew brainstem at 15 min post ZD7288 treatment (1 mg/kg, i. p.). Moreover, the ERK1/2 inhibitor U0126 (20 mg/kg, i. p.), dose-dependently reduced the frequency of ZD7288-induced vomiting, suggesting the involvement of ERK1/2 signaling in the emesis evoked by HCN channel blocker ZD7288.

### Effects of Diverse Antiemetics on ZD7288-Induced Vomiting

#### LTCC Inhibitor Nifedipine

Ca^2+^ is regarded as the final intracellular messenger in the process of muscle excitation-contraction coupling, and blockade of Ca^2+^ entry through LTCCs prevents muscle contraction (Godfraind et al., 1986). The LTCC antagonist nifedipine prevents vomiting in a potent and dose-dependent manner when evoked by a wide range of emetogens including agonists of LTCC (FPL64176)-, serotonin 5-HT_3_- (e.g., 5-HT or 2-Methyl-5-HT)-, neurokinin NK_1_- (GR73632)-, dopamine D_2/3_- (apomorphine or quinpirole)-, and muscarinic M_1_- (McN-A-343)-receptors (Darmani et al., 2014b); as well as the sarcoplasmic endoplasmic reticulum calcium ATPase (SERCA) inhibitor thapsigargin (Zhong et al., 2016). Per our recent review (Zhong et al., 2017), the mechanism underlying broadspectrum antiemetic potential of nifedipine against these diverse emetogens is closely related to mobilization of Ca^2+^. In the current study, a 5 mg/kg (s.c.) dose of nifedipine offered significant, and at 10 mg/kg exerted complete inhibition of vomiting evoked by ZD7288 (1 mg/kg, i. p.). At this dosage range, nifedipine can also protect vomiting evoked by the forementioned emetogens (Darmani et al., 2014b). Thus, these findings demonstrate that ZD7288-induced vomiting is highly sensitive to the LTCC blocker nifedipine.

#### TRPV1R Agonist Resiniferatoxin

It is interesting to note that HCN channels exhibit structural similarities to TRPV1R with different gated mechanisms (Gill et al., 2004). Capsazepine, a well-known inhibitor of TRPV1R, possess HCN1 blocking activity (Biel et al., 2009). Our unpublished data show capsazepine (5 mg/kg, i. p.) is pro-emetic in the least shrews. Our previous studies have shown that the selective and ultra-potent TRPV1R agonist RTX has pro and antiemetic effects in the least shrew model of emesis (Darmani et al., 2014a; Darmani et al., 2020). A subcutaneous injection of RTX by itself induces vomiting in the least shrew at doses higher than 10 μg/kg. Lower (0.01–5.0 μg/kg) doses of RTX can suppress vomiting induced by diverse emetogens (Darmani et al., 2020). Here we demonstrate that RTX at 2.5 μg/kg completely abolishes vomiting caused by ZD7288, which further supports the potent broad-spectrum antiemetic potential of RTX, which also has been demonstrated by several other groups (Andrews et al., 2000; Yamakuni et al., 2002; Darmani et al., 2014a; Rudd et al., 2015; Aghazadeh Tabrizi et al., 2017). The TRPV1R is expressed on neuronal membrane as well as in the membrane of intracellular organelles and plays a crucial role in maintaining intracellular Ca^2+^ homeostasis (Lang et al., 2015; Hurt et al., 2016; Zhao and Tsang, 2017). Mechanisms of antiemetic effects of RTX are still unclear, but probably involves inhibition of voltage activated Ca^2+^ channels (Andrews et al., 2000) as well as desensitization of vagal afferents and its terminals in the NTS (Shiroshita et al., 1997).

#### SOCE Inhibitor MRS 1845

Store-operated Ca^2+^ entry (SOCE) at the plasma membrane can be activated following Ca^2+^ release from intracellular Ca^2+^ stores through IP_3_R- and/or RyR-channels in both non-excitable and excitable cells (Putney and McKay, 1999; Putney, 2003; Parekh and Putney, 2005). In the current study, we found that relative to complete suppression of ZD7288-evoked vomiting by nifedipine at 10 mg/kg dose, pretreatment with MRS 1845, a potent and selective SOCE inhibitor, at the same dosage, significantly but partially reduced the frequency of the ZD7288-evoked vomiting without providing complete emesis protection in any of tested shrew (*p* > 0.05). These findings implicate a significant contribution of LTCC-mediated extracellular Ca^2+^ influx and a minor role for SOCE in ZD7288-induced vomiting. MRS 1845 at the same dosage has also been shown to in part reduce the frequency of GR73632-evoked vomiting without providing complete emesis protection (*p* > 0.05) (Zhong et al., 2019). It also failed to exert a significant impact on thapsigargin-evoked vomiting (Zhong et al., 2016), which further supports a minor role for SOCE in vomiting.

#### T-type Ca^2+^ Channel Inhibitor

Like the SOCE inhibitor MRS 1845, the potent and selective T-type Ca^2+^ channel blocker Z944 at the same dose range, significantly attenuated the frequency of ZD7288-evoked vomiting without providing complete emesis protection (*p* > 0.05), suggesting a minor role for T-type Ca^2+^ channel in mechanisms underlying ZD7288-induced vomiting. Presynaptic HCN channels and T-type Ca^2+^ channels are present on the same neuronal terminals and play a role in neurotransmitter release (Gill et al., 2004). Presynaptic HCN channels pharmacological blockade or genetic ablation of HCN1 channels, can lead to membrane hyperpolarization and enhanced Ca^2+^ influx through T-type Ca^2+^ channels, boosting spontaneous synaptic release (Huang et al., 2011; Shah, 2014). Therefore, it is possible that the minor antiemetic effect of T-type Ca^2+^ channel blocker Z944 is due to limiting presynaptic neurotransmitter release evoked by the HCN channel blocker ZD7288 (Klar et al., 2003; Tokay et al., 2009). This hypothesis needs further investigation.

#### Intracellular Ca^2+^ Channel Inhibitors

We have previously shown that intracellular sarcoplasmic/endoplasmic reticulum luminal Ca^2+^ release channels RyRs and IP_3_Rs can be differentially modulated by diverse emetogens including 2-Methyl-5-HT (Zhong et al., 2014), thapsigargin (Zhong et al., 2016) and the NK_1_ receptor agonist GR73632 (Zhong et al., 2019). Here, we also demonstrate that significant reductions (60–85%) in the frequency of ZD7288-induced vomiting (but without full emesis protection) occur when shrews are pretreated with inhibitors of either RyRs (dantrolene at 5–10 mg/kg) or IP_3_Rs (2-APB at 10 mg/kg). Moreover, while a mixture of dantrolene (1 mg/kg) and 2-APB (1 mg/kg) did offer a slightly additional protection beyond what was afforded when each drug was administered alone, a mixture of the latter doses of 2-APB plus dantrolene combined with a 1 mg/kg dose of nifedipine, led to a further reduction in ZD7288-evoked vomiting (Data not shown). However, none of these formulations provided complete protection against ZD7288-induced vomiting (Data not shown). On the contrary, we have previously shown that combined pretreatment with low doses of nifidipine, dantrolene and 2-APB completely protects shrews from thapsigargin-evoked emesis (Zhong et al., 2016). Our present study provides *in vivo* evidence that the HCN blocker ZD7288 causes vomiting, a process that is partially dependent upon Ca^2+^ mobilization which may involve Ca^2+^ intracellular store release through IP_3_Rs and RyRs, as well as extracellular Ca^2+^ entry via plasma membrane Ca^2+^ channels (such as LTCC, SOCE, T-type Ca^2+^ channel).

#### Effects of Diverse Emetic Receptor Antagonists

Substantial evidence indicates that cancer cytotoxic chemotherapeutic drugs including cisplatin cause vomiting via indirect stimulation of serotonin 5-HT_3_R, neurokinin NK_1_R and dopamine D_2_R, subsequent to release of corresponding monoamines in both the brainstem and gastrointestinal tract emetic loci (Darmani et al., 2009; Darmani and Ray, 2009). In the current study, we investigated the possibility of involvement of 5-HT_3_R, NK_1_R and D_2_R in vomiting evoked by the HCN channel blocker ZD7288. Indeed, the selective 5-HT_3_ receptor antagonist palonosetron at 0.5 mg/kg significantly attenuated the vomit frequency evoked by ZD7288 by 50% but failed to completely protect shrews from vomiting up to a 5 mg/kg dose. Moreover, at the same dose-range, palonosetron has been shown not to affect vomiting in least shrews evoked either by the LTCC activator FPL64176 (Darmani et al., 2014b) or the intracellular Ca^2+^ releaser, thapsigargin (Zhong et al., 2016). In fact, 5-HT_3_ antagonists are considered as narrow-spectrum antiemetics (Sanger and Andrews, 2018). Netupitant and other neurokinin NK_1_R antagonists possess broad-spectrum antiemetic efficacy against several emetic challenges including cisplatin, apomorphine, morphine, ipecacuanha, copper sulfate and motion-induced emesis (Rudd and Andrews, 2004; Darmani et al., 2015; Rudd et al., 2016). In the present study, netupitant (10 mg/kg, i. p.) exerted 85% reduction in the frequency of ZD7288-induced emesis, but without providing complete emesis protection against the evoked vomiting. On the other hand, at this dose netupitant has been shown to provide a significant and near complete protection against vomiting evoked by thapsigargin (Zhong et al., 2016) and complete protection against vomiting evoked the NK_1_R agonist GR73632 (5 mg/kg, i. p.) in least shrews (Zhong et al., 2019). In comparison, the D_2/3_R antagonist sulpride at 8 mg/kg dose failed to affect ZD7288-induced emesis. This dose of sulpride can protect up to 80% of shrews from vomiting caused by D_2_R preferring agonist quinpirole, and its 2 mg/kg dose can fully protect shrews against vomiting caused by the nonselective dopamine receptor agonist apomorphine (Darmani et al., 1999).

## Conclusion

Taken together, our findings demonstrate that the HCN channel blocker ZD7288, is a potent emetogen in least shrews. In fact, not only ZD7288 induces vomiting rapidly and in a potent manner, but except for nifedipine and resiniferatoxin, none of the other well-known tested antiemetics could provide total protection against the evoked vomiting. The induced emetic behavior was accompanied by a major central contribution as indicated by the ZD7288-evoked expression of c-Fos and ERK1/2 phosphorylation in the brainstem DVC emetic nuclei. Furthermore, ZD7288 induced c-Fos expression occurred in TPH2 positive neurons of the brainstem DVC emetic nuclei, suggesting activation of serotonin neurons may contribute to the evoked vomiting.

## Data Availability

The raw data supporting the conclusions of this article will be made available by the authors, without undue reservation.

## Supplementary Material

Supplemental Figure 1 | Quantified data for ZD7288-induced c-Fos expression in TPH2 positive cells in the least shrew brainstem dorsal vagal complex, containing the area postrema (AP), the nucleus tractus solitarius (NTS) and the dorsal motor nucleus of the vagus (DMNX). Values represent the mean number of c-Fos expressing and TPH2 positive cells in each region of interest per section and are presented as mean ± SEM (n = 3 shrews per group). ***p* < 0.01, ****p* < 0.001 vs. Control (treated with vehicle of ZD7288), Unpaired t-test.

## Abbreviations

HCN, Hyperpolarization-activated cyclic nucleotide-gated; DVC, Dorsal vagal complex; AP, Area postrema; NTS, Nucleus tractus solitarius; DMNX, Dorsal motor nucleus of the vagus; ENS, Enteric nervous system; TPH2, Tryptophan hydroxylase 2; LTCC, L-type Ca2+ channel; SOCE, Store-operated Ca2+ entry; TRPV1R, Transient receptor potential vanilloid receptor 1; RTX, Resiniferatoxin; RyR, Ryanodine receptor; IP3R, Inositol-1, 4, 5-trisphosphate receptor; 5-HT3R, Serotonergic type 3 receptor; NK1R, Neurokinin 1 receptor; D2R, Dopamine type 2 receptor; i.p., Intraperitoneal; s.c., Subcutaneously; i.c.v., Intracerebroventricular; pERK1/2, Phospho-ERK1/2.

## References

[B1] Adel N. (2017). Overview of Chemotherapy-Induced Nausea and Vomiting and Evidence-Based Therapies. Am. J. Manag. Care 23, S259–S265. 28978206

[B2] Aghazadeh Tabrizi M. Baraldi P. G. Baraldi S. Gessi S. Merighi S. Borea P. A. (2017). Medicinal Chemistry, Pharmacology, and Clinical Implications of TRPV1 Receptor Antagonists. Med. Res. Rev. 37, 936–983. 10.1002/med.21427 27976413

[B3] Andrews P. L. Okada F. Woods A. J. Hagiwara H. Kakaimoto S. Toyoda M. (2000). The Emetic and Anti-emetic Effects of the Capsaicin Analogue Resiniferatoxin in Suncus Murinus, the House Musk Shrew. Br. J. Pharmacol. 130, 1247–1254. 10.1038/sj.bjp.0703428 10903962 PMC1572188

[B4] Babic T. Browning K. N. (2014). The Role of Vagal Neurocircuits in the Regulation of Nausea and Vomiting. Eur. J. Pharmacol. 722, 38–47. 10.1016/j.ejphar.2013.08.047 24184670 PMC3893663

[B5] Beleslin D. B. Krstić S. K. Stefanović-Denić K. Strbac M. Mićić D. (1981). Inhibition by Morphine and Morphine-like Drugs of Nicotine-Induced Emesis in Cats. Brain Res. Bull. 6, 451–453. 10.1016/s0361-9230(81)80017-2 7248811

[B6] Benarroch E. E. (2013). HCN Channels: Function and Clinical Implications. Neurology 80, 304–310. 10.1212/WNL.0b013e31827dec42 23319474

[B7] Bhandari P. Bingham S. Andrews P. L. (1992). The Neuropharmacology of Loperamide-Induced Emesis in the Ferret: the Role of the Area Postrema, Vagus, Opiate and 5-HT3 Receptors. Neuropharmacology 31, 735–742. 10.1016/0028-3908(92)90034-m 1326727

[B8] Biel M. Wahl-Schott C. Michalakis S. Zong X. (2009). Hyperpolarization-activated Cation Channels: from Genes to Function. Physiol. Rev. 89, 847–885. 10.1152/physrev.00029.2008 19584315

[B9] Borison H. L. (1989). Area Postrema: Chemoreceptor Circumventricular Organ of the Medulla Oblongata. Prog. Neurobiol. 32, 351–390. 10.1016/0301-0082(89)90028-2 2660187

[B10] Bucchi A. Baruscotti M. Nardini M. Barbuti A. Micheloni S. Bolognesi M. (2013). Identification of the Molecular Site of Ivabradine Binding to HCN4 Channels. PLoS One 8, e53132. 10.1371/journal.pone.0053132 23308150 PMC3537762

[B11] Bullitt E. (1990). Expression of C-fos-like Protein as a Marker for Neuronal Activity Following Noxious Stimulation in the Rat. J. Comp. Neurol. 296, 517–530. 10.1002/cne.902960402 2113539

[B12] Cai Y. Q. Wang W. Hou Y. Y. Pan Z. Z. (2014). Optogenetic Activation of Brainstem Serotonergic Neurons Induces Persistent Pain Sensitization. Mol. Pain 10, 70. 10.1186/1744-8069-10-70 25410898 PMC4247651

[B13] Casillas-Espinosa P. M. Hicks A. Jeffreys A. Snutch T. P. O'Brien T. J. Powell K. L. (2015). Z944, a Novel Selective T-type Calcium Channel Antagonist Delays the Progression of Seizures in the Amygdala Kindling Model. PLoS One 10, e0130012. 10.1371/journal.pone.0130012 26274319 PMC4537250

[B14] Chang W. T. Gao Z. H. Li S. W. Liu P. Y. Lo Y. C. Wu S. N. (2020). Characterization in Dual Activation by Oxaliplatin, a Platinum-Based Chemotherapeutic Agent of Hyperpolarization-Activated Cation and Electroporation-Induced Currents. Int. J. Mol. Sci. 21, 396. 10.3390/ijms21020396 PMC7014111 31936301

[B15] Chaplan S. R. Guo H. Q. Lee D. H. Luo L. Liu C. Kuei C. (2003). Neuronal Hyperpolarization-Activated Pacemaker Channels Drive Neuropathic Pain. J. Neurosci. 23, 1169–1178. 10.1523/JNEUROSCI.23-04-01169.2003 12598605 PMC6742242

[B16] Chaturvedi A. Singh Y. Chaturvedi H. Thawani V. Singla S. Parihar D. (2013). Comparison of the Efficacy and Tolerability of Ivabradine and Ranolazine in Patients of Chronic Stable Angina Pectoris. J. Pharm. Pharmacother. 4, 33–38. 10.4103/0976-500X.107663 PMC3643340 23662022

[B17] Chebolu S. Wang Y. Ray A. P. Darmani N. A. (2010). Pranlukast Prevents Cysteinyl Leukotriene-Induced Emesis in the Least Shrew (Cryptotis Parva). Eur. J. Pharmacol. 628, 195–201. 10.1016/j.ejphar.2009.11.030 19941848 PMC2818547

[B18] Chen X. Shu S. Kennedy D. P. Willcox S. C. Bayliss D. A. (2009). Subunit-specific Effects of Isoflurane on Neuronal Ih in HCN1 Knockout Mice. J. Neurophysiol. 101, 129–140. 10.1152/jn.01352.2007 18971302 PMC2637007

[B19] Darmani N. A. Chebolu S. Zhong W. Trinh C. McClanahan B. Brar R. S. (2014a). Additive Antiemetic Efficacy of Low-Doses of the Cannabinoid CB(1/2) Receptor Agonist δ(9)-THC with Ultralow-Doses of the Vanilloid TRPV1 Receptor Agonist Resiniferatoxin in the Least Shrew (Cryptotis Parva). Eur. J. Pharmacol. 722, 147–155. 10.1016/j.ejphar.2013.08.051 24157976

[B20] Darmani N. A. Crim J. L. Janoyan J. J. Abad J. Ramirez J. (2009). A Re-evaluation of the Neurotransmitter Basis of Chemotherapy-Induced Immediate and Delayed Vomiting: Evidence from the Least Shrew. Brain Res. 1248, 40–58. 10.1016/j.brainres.2008.10.063 19022231

[B21] Darmani N. A. Henry D. A. Zhong W. Chebolu S. (2020). Ultra-low Doses of the Transient Receptor Potential Vanilloid 1 Agonist, Resiniferatoxin, Prevents Vomiting Evoked by Diverse Emetogens in the Least Shrew (Cryptotis Parva). Behav. Pharmacol. 31, 3–14. 10.1097/FBP.0000000000000499 31503071 PMC6954338

[B22] Darmani N. A. Mock O. B. Towns L. C. Gerdes C. F. (1994). The Head-Twitch Response in the Least Shrew (Cryptotis Parva) Is a 5-HT_2_- and Not a 5-HT_1_C-Mediated Phenomenon. Pharmacol. Biochem. Behav. 48, 383–396. 10.1016/0091-3057(94)90542-8 8090805

[B23] Darmani N. A. Ray A. P. (2009). Evidence for a Re-evaluation of the Neurochemical and Anatomical Bases of Chemotherapy-Induced Vomiting. Chem. Rev. 109, 3158–3199. 10.1021/cr900117p 19522506

[B24] Darmani N. A. (1998). Serotonin 5-HT_3_ Receptor Antagonists Prevent Cisplatin-Induced Emesis in Cryptotis Parva: a New Experimental Model of Emesis. J. Neural Transm. 105, 1143–1154. 10.1007/s007020050118 9928884

[B25] Darmani N. A. Wang Y. Abad J. Ray A. P. Thrush G. R. Ramirez J. (2008). Utilization of the Least Shrew as a Rapid and Selective Screening Model for the Antiemetic Potential and Brain Penetration of Substance P and NK_1_ Receptor Antagonists. Brain Res. 1214, 58–72. 10.1016/j.brainres.2008.03.077 18471804 PMC2486262

[B26] Darmani N. A. Zhao W. Ahmad B. (1999). The Role of D2 and D3 Dopamine Receptors in the Mediation of Emesis in Cryptotis Parva (The Least Shrew). J. Neural Transm. 106, 1045–1061. 10.1007/s007020050222 10651102

[B27] Darmani N. A. Zhong W. Chebolu S. Mercadante F. (2015). Differential and Additive Suppressive Effects of 5-HT_3_ (Palonosetron)- and NK_1_ (Netupitant)-receptor Antagonists on Cisplatin-Induced Vomiting and ERK1/2, PKA and PKC Activation. Pharmacol. Biochem. Behav. 131, 104–111. 10.1016/j.pbb.2015.02.010 25687374

[B28] Darmani N. A. Zhong W. Chebolu S. Vaezi M. Alkam T. (2014b). Broad-spectrum Antiemetic Potential of the L-type Calcium Channel Antagonist Nifedipine and Evidence for its Additive Antiemetic Interaction with the 5-HT(3) Receptor Antagonist Palonosetron in the Least Shrew (*Cryptotis Parva*). Eur. J. Pharmacol. 722, 2–12. 10.1016/j.ejphar.2013.08.052 24513517

[B29] Ding W. You Z. Shen S. Yang J. Lim G. Doheny J. T. (2018). Increased HCN Channel Activity in the Gasserian Ganglion Contributes to Trigeminal Neuropathic Pain. J. Pain. 19, 626–634. 10.1016/j.jpain.2018.01.003 29366880 PMC5972061

[B30] Doan T. N. Stephans K. Ramirez A. N. Glazebrook P. A. Andresen M. C. Kunze D. L. (2004). Differential Distribution and Function of Hyperpolarization-Activated Channels in Sensory Neurons and Mechanosensitive Fibers. J. Neurosci. 24, 3335–3343. 10.1523/JNEUROSCI.5156-03.2004 15056713 PMC6730026

[B31] Felix R. Sandoval A. Sánchez D. Gómora J. C. De la Vega-Beltrán J. L. Treviño C. L. (2003). ZD7288 Inhibits Low-Threshold Ca 2+ Channel Activity and Regulates Sperm Function. Biochem. Biophys. Res. Commun. 311, 187–192. 10.1016/j.bbrc.2003.09.197 14575712

[B32] Ferrini F. Russo A. Salio C. (2014). Fos and pERK Immunoreactivity in Spinal Cord Slices: Comparative Analysis of In Vitro Models for Testing Putative Antinociceptive Molecules. Ann. Anat. 196, 217–223. 10.1016/j.aanat.2013.11.005 24447791

[B33] Funahashi M. Mitoh Y. Matsuo R. (2004). The Sensitivity of Hyperpolarization-Activated Cation Current (Ih) to Propofol in Rat Area Postrema Neurons. Brain Res. 1015, 198–201. 10.1016/j.brainres.2004.04.043 15223387

[B34] Funahashi M. Mitohm Y. Kohjitani A. Matsuo R. (2003). Role of the Hyperpolarization-Activated Cation Current (Ih) in Pacemaker Activity in Area Postrema Neurons of Rat Brain Slices. J. Physiol. 552, 135–148. 10.1113/jphysiol.2003.047191 12897173 PMC2343317

[B35] Furness J. B. (2000). Types of Neurons in the Enteric Nervous System. J. Auton. Nerv. Syst. 81, 87–96. 10.1016/s0165-1838(00)00127-2 10869706

[B36] Gill C. H. Randall A. Bates S. A. Hill K. Owen D. Larkman P. M. (2004). Characterization of the Human HCN1 Channel and its Inhibition by Capsazepine. Br. J. Pharmacol. 143, 411–421. 10.1038/sj.bjp.0705945 15351778 PMC1575350

[B37] Godfraind T. Miller R. Wibo M. (1986). Calcium Antagonism and Calcium Entry Blockade. Pharmacol. Rev. 38, 321–416. 2432624

[B38] Griguoli M. Maul A. Nguyen C. Giorgetti A. Carloni P. Cherubini E. (2010). Nicotine Blocks the Hyperpolarization-Activated Current Ih and Severely Impairs the Oscillatory Behavior of Oriens-Lacunosum Moleculare Interneurons. J. Neurosci. 30, 10773–10783. 10.1523/JNEUROSCI.2446-10.2010 20702707 PMC6634696

[B39] He J. T. Li X. Y. Zhao X. Liu X. (2019). Hyperpolarization-activated and Cyclic Nucleotide-Gated Channel Proteins as Emerging New Targets in Neuropathic Pain. Rev. Neurosci. 30, 639–649. 10.1515/revneuro-2018-0094 30768426

[B40] Herrmann S. Schnorr S. Ludwig A. (2015). HCN Channels-Mmodulators of Cardiac and Neuronal Excitability. Int. J. Mol. Sci. 16, 1429–1447. 10.3390/ijms16011429 25580535 PMC4307311

[B41] Horn C. C. Meyers K. Oberlies N. (2014). Musk Shrews Selectively Bred for Motion Sickness Display Increased Anesthesia-Induced Vomiting. Physiol. Behav. 124, 129–137. 10.1016/j.physbeh.2013.11.002 24239993 PMC3887396

[B42] Hotokezaka H. Sakai E. Kanaoka K. Saito K. Matsuo K. Kitaura H. (2002). U0126 and PD98059, Specific Inhibitors of MEK, Accelerate Differentiation of RAW264.7 Cells into Osteoclast-like Cells. J. Biol. Chem. 277, 47366–47372. 10.1074/jbc.M208284200 12237315

[B43] Huang Z. Lujan R. Kadurin I. Uebele V. N. Renger J. J. Dolphin A. C. (2011). Presynaptic HCN1 Channels Regulate Cav3.2 Activity and Neurotransmission at Select Cortical Synapses. Nat. Neurosci. 14, 478–486. 10.1038/nn.2757 21358644 PMC3068302

[B44] Hurt C. M. Lu Y. Stary C. M. Piplani H. Small B. A. Urban T. J. (2016). Transient Receptor Potential Vanilloid 1 Regulates Mitochondrial Membrane Potential and Myocardial Reperfusion Injury. J. Am. Heart Assoc. 5, e003774. 10.1161/JAHA.116.003774 27671317 PMC5079036

[B45] Johard H. Omelyanenko A. Fei G. Zilberter M. Dave Z. Abu-Youssef R. (2020). HCN Channel Activity Balances Quiescence and Proliferation in Neural Stem Cells and Is a Selective Target for Neuroprotection During Cancer Treatment. Mol. Cancer Res. 18, 1522–1533. 10.1158/1541-7786.MCR-20-0292 32665429

[B46] Kharouf Q. Phillips A. M. Bleakley L. E. Morrisroe E. Oyrer J. Jia L. (2020a). The Hyperpolarization-Activated Cyclic Nucleotide-Gated 4 Channel as a Potential Anti-seizure Drug Target. Br. J. Pharmacol. 177, 3712–3729. 10.1111/bph.15088 32364262 PMC7393203

[B47] Kharouf Q. Pinares-Garcia P. Romanelli M. N. Reid C. A. (2020b). Testing Broad-Spectrum and Isoform-Preferring HCN Channel Blockers for Anticonvulsant Properties in Mice. Epilepsy Res. 168, 106484. 10.1016/j.eplepsyres.2020.106484 33099130

[B48] Kitayama M. Miyata H. Yano M. Saito N. Matsuda Y. Yamauchi T. (2003). Ih Blockers Have a Potential of Antiepileptic Effects. Epilepsia 44, 20–24. 10.1046/j.1528-1157.2003.22702.x 12581225

[B49] Klar M. Surges R. Feuerstein T. J. (2003). Ih Channels as Modulators of Presynaptic Terminal Function: ZD7288 Increases NMDA-Evoked [3H]-Noradrenaline Release in Rat Neocortex Slices. Naunyn Schmiedebergs Arch. Pharmacol. 367, 422–425. 10.1007/s00210-003-0707-6 12690435

[B50] Kodirov S. A. Wehrmeister M. Colom L. V. (2014). Modulation of HCN Channels in Lateral Septum by Nicotine. Neuropharmacology 81, 274–282. 10.1016/j.neuropharm.2014.02.012 24582613 PMC5384640

[B51] Lang H. Yu H. Li P. Lu Z. Xiong S. (2015). Activation of TRPV1 Attenuates High Salt-Induced Cardiac Hypertrophy through Improvement of Mitochondrial Function. Br. J. Pharmacol. 172, 5548–5558. 10.1111/bph.12987 25339153 PMC4667858

[B52] Lee D. H. Chang L. Sorkin L. S. Chaplan S. R. (2005). Hyperpolarization-activated, Cation-Nonselective, Cyclic Nucleotide-Modulated Channel Blockade Alleviates Mechanical Allodynia and Suppresses Ectopic Discharge in Spinal Nerve Ligated Rats. J. Pain. 6, 417–424. 10.1016/j.jpain.2005.02.002 15993819

[B53] Lee Y. T. Vasilyev D. V. Shan Q. J. Dunlop J. Mayer S. Bowlby M. R. (2008). Novel Pharmacological Activity of Loperamide and CP-339,818 on Human HCN Channels Characterized with an Automated Electrophysiology Assay. Eur. J. Pharmacol. 581, 97–104. 10.1016/j.ejphar.2007.11.058 18162181

[B54] Li X. Chen W. Zhang L. Liu W. B. Fei Z. (2013). Inhibition of Store-Operated Calcium Entry Attenuates MPP(+)-induced Oxidative Stress via Preservation of Mitochondrial Function in PC12 Cells: Involvement of Homer1a. PLoS One 8, e83638. 10.1371/journal.pone.0083638 24358303 PMC3866123

[B55] Luo L. Chang L. Brown S. M. Ao H. Lee D. H. Higuera E. S. (2007). Role of Peripheral Hyperpolarization-Activated Cyclic Nucleotide-Modulated Channel Pacemaker Channels in Acute and Chronic Pain Models in the Rat. Neuroscience 144, 1477–1485. 10.1016/j.neuroscience.2006.10.048 17196750

[B56] McGovern A. E. Robusto J. Rakoczy J. Simmons D. G. Phipps S. Mazzone S. B. (2014). The Effect of Hyperpolarization-Activated Cyclic Nucleotide-Gated Ion Channel Inhibitors on the Vagal Control of guinea Pig Airway Smooth Muscle Tone. Br. J. Pharmacol. 171, 3633–3350. 10.1111/bph.12745 24762027 PMC4128062

[B57] Meng Q. T. Xia Z. Y. Liu J. Bayliss D. A. Chen X. (2011). Local Anesthetic Inhibits Hyperpolarization-Activated Cationic Currents. Mol. Pharmacol. 79, 866–873. 10.1124/mol.110.070227 21303986 PMC3082936

[B58] Miller A. D. Leslie R. A. (1994). The Area Postrema and Vomiting. Front. Neuroendocrinol. 15, 301–320. 10.1006/frne.1994.1012 7895890

[B59] Milligan C. J. Edwards I. J. Deuchars J. (2006). HCN1 Ion Channel Immunoreactivity in Spinal Cord and Medulla Oblongata. Brain Res. 1081, 79–91. 10.1016/j.brainres.2006.01.019 16503331

[B60] Mizuta K. Gallos G. Zhu D. Mizuta F. Goubaeva F. Xu D. (2008). Expression and Coupling of Neurokinin Receptor Subtypes to Inositol Phosphate and Calcium Signaling Pathways in Human Airway Smooth Muscle Cells. Am. J. Physiol. Lung Cell Mol. Physiol. 294, L523–L534. 10.1152/ajplung.00328.2007 18203813 PMC3650481

[B61] Monteggia L. M. Eisch A. J. Tang M. D. Kaczmarek L. K. Nestler E. J. (2000). Cloning and Localization of the Hyperpolarization-Activated Cyclic Nucleotide-Gated Channel Family in Rat Brain. Brain Res. Mol. Brain Res. 81, 129–139. 10.1016/s0169-328x(00)00155-8 11000485

[B62] Navari R. M. Aapro M. (2016). Antiemetic Prophylaxis for Chemotherapy-Induced Nausea and Vomiting. N. Engl. J. Med. 374, 1356–1367. 10.1056/NEJMra1515442 27050207

[B63] Navari R. M. (2013). Management of Chemotherapy-Induced Nausea and Vomiting: Focus on Newer Agents and New Uses for Older Agents. Drugs 73, 249–262. 10.1007/s40265-013-0019-1 23404093

[B64] Ng L. C. Wilson S. M. McAllister C. E. Hume J. R. (2007). Role of InsP3 and Ryanodine Receptors in the Activation of Capacitative Ca2+ Entry by Store Depletion or Hypoxia in Canine Pulmonary Arterial Smooth Muscle Cells. Br. J. Pharmacol. 152, 101–111. 10.1038/sj.bjp.0707357 17592501 PMC1978272

[B65] Novella Romanelli M. Sartiani L. Masi A. Mannaioni G. Manetti D. Mugelli A. (2016). HCN Channels Modulators: The Need for Selectivity. Curr. Top. Med. Chem. 16, 1764–1791. 10.2174/1568026616999160315130832 26975509 PMC5374843

[B66] Okura M. Riehl J. Mignot E. Nishino S. (2000). Sulpiride, a D2/D3 Blocker, Reduces Cataplexy but Not REM Sleep in Canine Narcolepsy. Neuropsychopharmacology 23, 528–538. 10.1016/S0893-133X(00)00140-8 11027918

[B67] Parekh A. B. Putney J. W. Jr. (2005). Store-operated Calcium Channels. Physiol. Rev. 85, 757–810. 10.1152/physrev.00057.2003 15788710

[B68] Parker L. A. Limebeer C. L. Rock E. M. Litt D. L. Kwiatkowska M. Piomelli D. (2009). The FAAH Inhibitor URB-597 Interferes with Cisplatin- and Nicotine-Induced Vomiting in the Suncus Murinus (House Musk Shrew). Physiol. Behav. 97, 121–124. 10.1016/j.physbeh.2009.02.014 19239915 PMC3781595

[B69] Peng T. Qi B. He J. Ke H. Shi J. (2020). Advances in the Development of Phosphodiesterase-4 Inhibitors. J. Med. Chem. 63, 10594–10617. 10.1021/acs.jmedchem.9b02170 32255344

[B70] Postea O. Biel M. (2011). Exploring HCN Channels as Novel Drug Targets. Nat. Rev. Drug Discov. 10, 903–914. 10.1038/nrd3576 22094868

[B71] Putney J. W. (2003). Capacitative Calcium Entry in the Nervous System. Cell Calcium 34, 339–344. 10.1016/s0143-4160(03)00143-x 12909080

[B72] Putney J. W. McKay R. R. (1999). Capacitative Calcium Entry Channels. Bioessays 21, 38–46. 10.1002/(SICI)1521-1878(199901)21:1<38::AID-BIES5>3.0.CO;2-S 10070252

[B73] Ramírez D. Zúñiga R. Concha G. Zúñiga L. (2018). HCN Channels: New Therapeutic Targets for Pain Treatment. Molecules 23, 2094. 10.3390/molecules23092094 PMC6225464 30134541

[B74] Ray A. P. Chebolu S. Darmani N. A. (2009a). Receptor-selective Agonists Induce Emesis and Fos Expression in the Brain and Enteric Nervous System of the Least Shrew (Cryptotis Parva). Pharmacol. Biochem. Behav. 94, 211–218. 10.1016/j.pbb.2009.08.010 19699757 PMC2771428

[B75] Ray A. P. Chebolu S. Ramirez J. Darmani N. A. (2009b). Ablation of Least Shrew Central Neurokinin NK1 Receptors Reduces GR73632-Induced Vomiting. Behav. Neurosci. 123, 701–706. 10.1037/a0015733 19485577 PMC2714262

[B76] Ray A. P. Darmani N. A. (2007). A Histologically Derived Stereotaxic Atlas and Substance P Immunohistochemistry in the Brain of the Least Shrew (*Cryptotis Parva*) Support its Role as a Model Organism for Behavioral and Pharmacological Research. Brain Res. 1156, 99–111. 10.1016/j.brainres.2007.04.061 17540350 PMC2730826

[B77] Rivolta I. Anna Binda A. Masi A. DiFrancesco J. C. (2020). Cardiac and Neuronal HCN Channelopathies. Pflugers Arch. 472, 931–951. 10.1007/s00424-020-02384-3 32424620

[B78] Rudd J. A. Andrews P. L. R. (2004). “Mechanisms of Acute, Delayed and Anticipatory Vomiting in Cancer and Cancer Treatment,” in Management of Nausea and Vomiting in Cancer and Cancer Treatment. Editor Hesketh P. J. (New York, NY: Jones and Barlett Publishers Inc.), 15–66.

[B79] Rudd J. A. Nalivaiko E. Matsuki N. Wan C. Andrews P. L. (2015). The Involvement of TRPV1 in Emesis and Anti-emesis. Temperature (Austin) 2, 258–276. 10.1080/23328940.2015.1043042 27227028 PMC4843889

[B80] Rudd J. A. Ngan M. P. Lu Z. Higgins G. A. Giuliano C. Lovati E. (2016). Profile of Antiemetic Activity of Netupitant Alone or in Combination with Palonosetron and Dexamethasone in Ferrets and Suncus Murinus (House Musk Shrew). Front. Pharmacol. 7, 263. 10.3389/fphar.2016.00263 27630563 PMC5005416

[B81] Sánchez-Alonso J. L. Halliwell J. V. Colino A. (2008). ZD 7288 Inhibits T-type Calcium Current in Rat Hippocampal Pyramidal Cells. Neurosci. Lett. 439, 275–280. 10.1016/j.neulet.2008.05.016 18534748

[B82] Sanger G. J. Andrews P. L. R. (2018). A History of Drug Discovery for Treatment of Nausea and Vomiting and the Implications for Future Research. Front. Pharmacol. 9, 913. 10.3389/fphar.2018.00913 30233361 PMC6131675

[B83] Sarocchi M. Arboscello E. Ghigliotti G. Murialdo R. Bighin C. Gualandi F. (2018). Ivabradine in Cancer Treatment-Related Left Ventricular Dysfunction. Chemotherapy 63, 315–320. 10.1159/000495576 30840967

[B84] Sasselli V. Pachnis V. Burns A. J. (2012). The Enteric Nervous System. Dev. Biol. 366, 64–73. 10.1016/j.ydbio.2012.01.012 22290331

[B85] Shah M. M. (2014). Cortical HCN Channels: Function, Trafficking and Plasticity. J. Physiol. 592, 2711–2719. 10.1113/jphysiol.2013.270058 24756635 PMC4104471

[B86] Shinpo K. Hirai Y. Maezawa H. Totsuka Y. Funahashi M. (2012). The Role of Area Postrema Neurons Expressing H-Channels in the Induction Mechanism of Nausea and Vomiting. Physiol. Behav. 107, 98–103. 10.1016/j.physbeh.2012.06.002 22722099

[B87] Shiroshita Y. Koga T. Fukuda H. (1997). Capsaicin in the 4th Ventricle Abolishes Retching and Transmission of Emetic Vagal Afferents to Solitary Nucleus Neurons. Eur. J. Pharmacol. 339, 183–192. 10.1016/s0014-2999(97)01370-8 9473134

[B88] Tokay T. Rohde M. Krabbe S. Rehberg M. Bender R. A. Köhling R. (2009). HCN1 Channels Constrain DHPG-Induced LTD at Hippocampal Schaffer collateral-CA1 Synapses. Learn. Mem. 16, 769–776. 10.1101/lm.1556009 19940037

[B89] Triggle D. J. (2007). Calcium Channel Antagonists: Clinical Uses-Ppast, Present and Future. Biochem. Pharmacol. 74, 1–9. 10.1016/j.bc.p.2007.01.016 17276408

[B90] Ueno S. Matsuki N. Saito H. (1987). Suncus Murinus: a New Experimental Model in Emesis Research. Life Sci. 41, 513–518. 10.1016/0024-3205(87)90229-3 3600192

[B91] Yamakuni H. Sawai-Nakayama H. Imazumi K. Maeda Y. Matsuo M. Manda T. (2002). Resiniferatoxin Antagonizes Cisplatin-Induced Emesis in Dogs and Ferrets. Eur. J. Pharmacol. 442, 273–278. 10.1016/s0014-2999(02)01541-8 12065081

[B92] Yamamoto K. Ngan M. P. Takeda N. Yamatodani A. Rudd J. A. (2004). Differential Activity of Drugs to Induce Emesis and pica Behavior in Suncus Murinus (House Musk Shrew) and Rats. Physiol. Behav. 83, 151–156. 10.1016/j.physbeh.2004.08.006 15501502

[B93] Zaman S. Woods A. J. Watson J. W. Reynolds D. J. Andrews P. L. (2000). The Effect of the NK1 Receptor Antagonist CP-99,994 on Emesis and C-Fos Protein Induction by Loperamide in the Ferret. Neuropharmacology 39, 316–323. 10.1016/s0028-3908(99)00113-6 10670427

[B94] Zhang X. X. Min X. C. Xu X. L. Zheng M. Guo L. J. (2016). ZD7288, a Selective Hyperpolarization-Activated Cyclic Nucleotide-Gated Channel Blocker, Inhibits Hippocampal Synaptic Plasticity. Neural Regen. Res. 11, 779–786. 10.4103/1673-5374.182705 27335562 PMC4904469

[B95] Zhao R. Tsang S. Y. (2017). Versatile Roles of Intracellularly Located TRPV1 Channel. J. Cell Physiol. 232, 1957–1965. 10.1002/jcp.25704 27891582

[B96] Zhong W. Chebolu S. Darmani N. A. (2016). Thapsigargin-induced Activation of Ca(2+)-CaMKII-ERK in Brainstem Contributes to Substance P Release and Induction of Emesis in the Least Shrew. Neuropharmacology 103, 195–210. 10.1016/j.neuropharm.2015.11.023 26631534

[B97] Zhong W. Chebolu S. Darmani N. A. (2018). Intracellular Emetic Signaling Evoked by the L-type Ca2+ Channel Agonist FPL64176 in the Least Shrew (Cryptotis Parva). Eur. J. Pharmacol. 834, 157–168. 10.1016/j.ejphar.2018.06.035 29966616 PMC6104632

[B98] Zhong W. Chebolu S. Darmani N. A. (2019). Intracellular Emetic Signaling Cascades by Which the Selective Neurokinin Type 1 Receptor (NK1R) Agonist GR73632 Evokes Vomiting in the Least Shrew (Cryptotis Parva). Neurochem. Int. 122, 106–119. 10.1016/j.neuint.2018.11.012 30453005 PMC6294657

[B99] Zhong W. Chebolu S. Darmani N. A. (2021). Central and Peripheral Emetic Loci Contribute to Vomiting Evoked by the Akt Inhibitor MK-2206 in the Least Shrew Model of Emesis. Eur. J. Pharmacol. 25, 174065. Online ahead of print. 10.1016/j.ejphar.2021.174065 PMC8085164 33775646

[B100] Zhong W. Darmani N. A. (2020). The Pivotal Role of Glycogen Synthase Kinase 3 (GSK-3) in Vomiting Evoked by Specific Emetogens in the Least Shrew (Cryptotis Parva). Neurochem. Int. 132, 104603. 10.1016/j.neuint.2019.104603 31738972 PMC6911829

[B101] Zhong W. Hutchinson T. E. Chebolu S. Darmani N. A. (2014). Serotonin 5-HT3 Receptor-Mediated Vomiting Occurs via the Activation of Ca2+/CaMKII-dependent ERK1/2 Signaling in the Least Shrew (Cryptotis Parva). PLoS One 9, e104718. 10.1371/journal.pone.0104718 25121483 PMC4133232

[B102] Zhong W. Picca A. J. Lee A. S. Darmani N. A. (2017). Ca2+ Signaling and Emesis: Recent Progress and New Perspectives. Auton. Neurosci. 202, 18–27. 10.1016/j.autneu.2016.07.006 27473611

