# Supplementary figures and images for: The HCN Channel Blocker ZD7288 Induces Emesis in the Least Shrew (Cryptotis parva)
Source: Front Pharmacol. 2021 Apr 29;12:647021. doi: 10.3389/fphar.2021.647021 (PMC8117105; doi:10.3389/fphar.2021.647021)

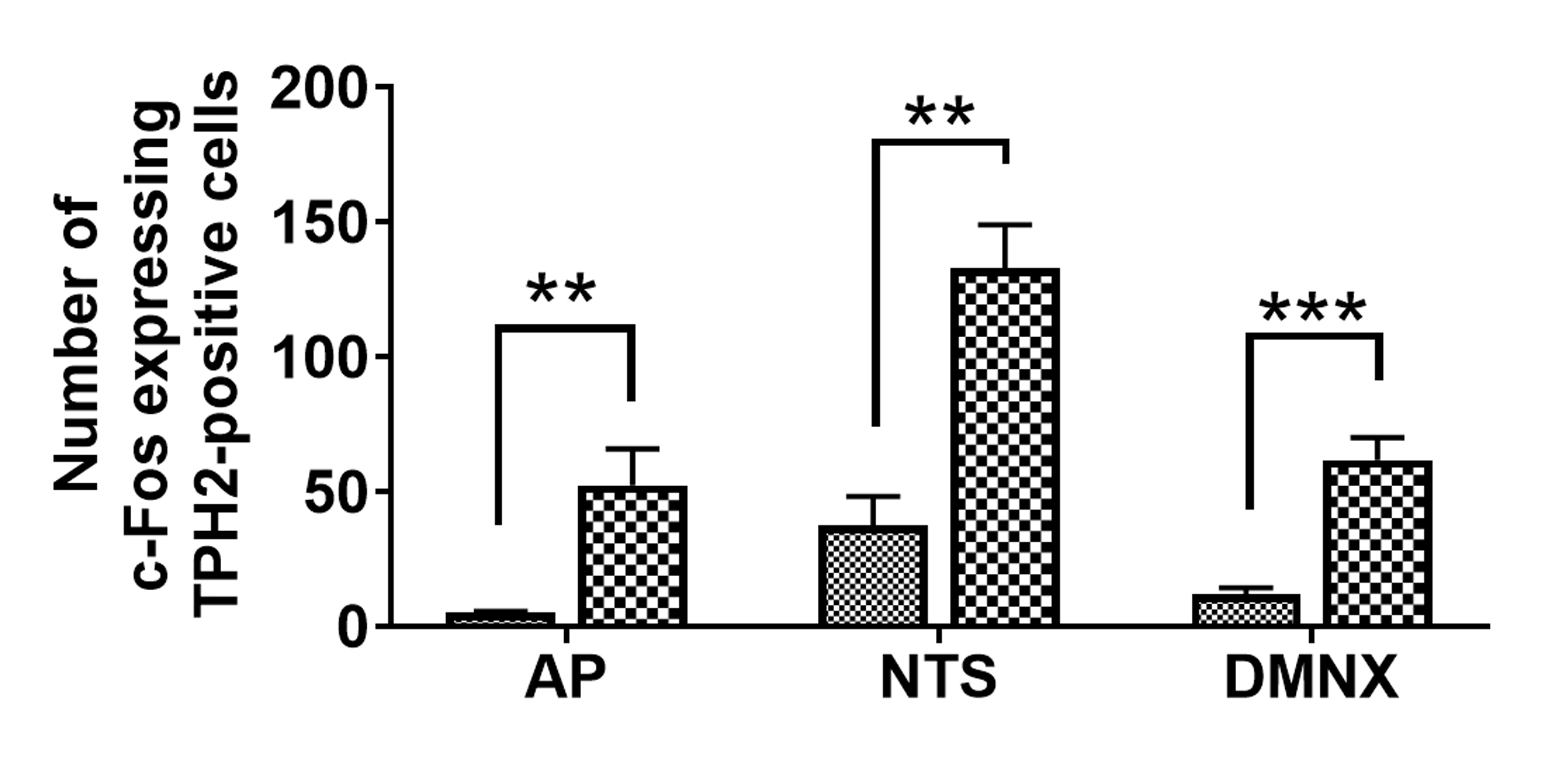

Supplement: Supplementary file 1 [file Image1.TIF]
